# DNA (de)methylation in embryonic stem cells controls CTCF-dependent chromatin boundaries

**DOI:** 10.1101/gr.239707.118

**Published:** 2019-05

**Authors:** Laura Wiehle, Graeme J. Thorn, Günter Raddatz, Christopher T. Clarkson, Karsten Rippe, Frank Lyko, Achim Breiling, Vladimir B. Teif

**Affiliations:** 1 Division of Epigenetics, DKFZ-ZMBH Alliance, German Cancer Research Center (DKFZ), 69120 Heidelberg, Germany;; 2 School of Biological Sciences, University of Essex, Wivenhoe Park, Colchester CO4 3SQ, United Kingdom;; 3 Division of Chromatin Networks, German Cancer Research Center (DKFZ) and Bioquant, 69120 Heidelberg, Germany

## Abstract

Coordinated changes of DNA (de)methylation, nucleosome positioning, and chromatin binding of the architectural protein CTCF play an important role for establishing cell-type–specific chromatin states during differentiation. To elucidate molecular mechanisms that link these processes, we studied the perturbed DNA modification landscape in mouse embryonic stem cells (ESCs) carrying a double knockout (DKO) of the *Tet1* and *Tet2* dioxygenases. These enzymes are responsible for the conversion of 5-methylcytosine (5mC) into its hydroxymethylated (5hmC), formylated (5fC), or carboxylated (5caC) forms. We determined changes in nucleosome positioning, CTCF binding, DNA methylation, and gene expression in DKO ESCs and developed biophysical models to predict differential CTCF binding. Methylation-sensitive nucleosome repositioning accounted for a significant portion of CTCF binding loss in DKO ESCs, whereas unmethylated and nucleosome-depleted CpG islands were enriched for CTCF sites that remained occupied. A number of CTCF sites also displayed direct correlations with the CpG modification state: CTCF was preferentially lost from sites that were marked with 5hmC in wild-type (WT) cells but not from 5fC-enriched sites. In addition, we found that some CTCF sites can act as bifurcation points defining the differential methylation landscape. CTCF loss from such sites, for example, at promoters, boundaries of chromatin loops, and topologically associated domains (TADs), was correlated with DNA methylation/demethylation spreading and can be linked to down-regulation of neighboring genes. Our results reveal a hierarchical interplay between cytosine modifications, nucleosome positions, and DNA sequence that determines differential CTCF binding and regulates gene expression.

Transcription factor (TF) binding and covalent DNA cytosine modifications like methylation (5mC), hydroxymethylation (5hmC), and formylation (5fC) occur in a cell-type–specific manner and are linked to the cellular gene expression program. Dependencies between DNA methylation and specific readers and effectors are well established (Schübeler 2015; Zhu et al. 2016). However, the molecular details of these interactions are often not well understood (Domcke et al. 2015; Yin et al. 2017). One important example of differential binding is the architectural protein CTCF that has functions in the direct regulation of transcription and the organization of 3D genome architecture (Merkenschlager and Nora 2016; Nora et al. 2017; Rao et al. 2017). A number of studies have linked differential CTCF binding to DNA (de)methylation (Stadler et al. 2011; Wang et al. 2012; Feldmann et al. 2013; Kasowski et al. 2013; Plasschaert et al. 2013; Teif et al. 2014; Maurano et al. 2015; Viner et al. 2016; Hashimoto et al. 2017), although in many cases, it remains unclear what is the cause and what is the consequence. About 40% of CTCF binding variability between different human cell types is correlated with DNA methylation changes (Wang et al. 2012). The methylation of a CTCF-dependent boundary element controlling imprinted expression of the *Igf2* gene has become a classical paradigm for the role of DNA methylation in reducing CTCF binding (Bell and Felsenfeld 2000). A similar effect of DNA methylation was reported for the *Dmpk* locus, in which deregulation of CTCF binding is linked to myotonic dystrophy (Filippova et al. 2001). DNA methylation can also decrease CTCF binding at intragenic sites involved in the regulation of splicing (Marina et al. 2016). However, not all CTCF binding sites contain CpG dinucleotides that can be methylated. In many cases, the causalities might be reverse: CTCF binding changes first and affects DNA methylation in the surrounding regions (Stadler et al. 2011; Maurano et al. 2015; Schübeler 2015). In the latter scenario, it remains largely unknown what determines the differences in CTCF binding in the first place. Previously, we proposed that a 5mC/5hmC/5fC switch can change the stability of nucleosomes at CTCF sites in a differentiation-dependent manner, thereby disturbing CTCF binding (Teif et al. 2014). Here, we used double-knockout (DKO) embryonic stem cells (ESCs) deficient for *Tet1* and *Tet2* (Dawlaty et al. 2013) to test this mechanism directly. TET1 and TET2 are responsible for the conversion of 5mC to 5hmC, 5fC, and 5caC (Tahiliani et al. 2009; Ito et al. 2011) and are required for ESC lineage specification (Koh et al. 2011). In DKO cells, 5hmC is absent (Dawlaty et al. 2013), which allowed us to investigate direct effects of 5hmC loss on the redistribution of 5mC, CTCF, and nucleosomes, as well as the corresponding changes in gene expression.

## Results

### Loss and gain of nucleosomes are linked to DNA methylation

**Figure 1.**
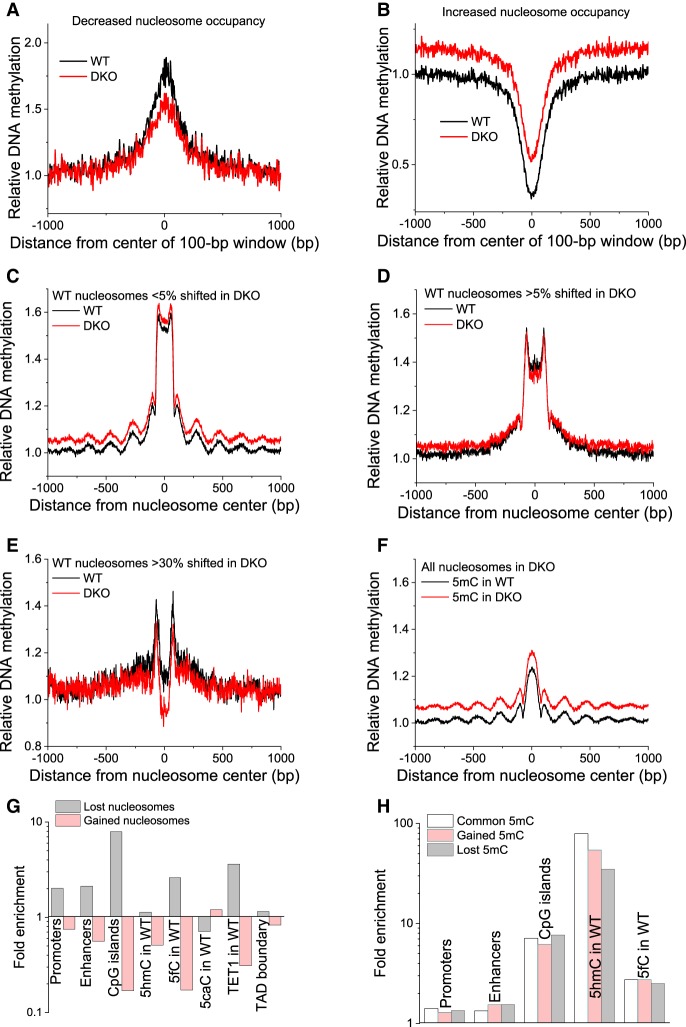
DNA methylation is associated with nucleosome repositioning. (*A*,*B*) Relative DNA methylation is shown around centers of 100-bp genomic regions with lost (*A*) and gained (*B*) nucleosome occupancy. (*C*–*F*) Changes in DNA methylation were associated with shifted nucleosomes. Relative DNA methylation is plotted as a function of the distance from the centers of nucleosomes on Chromosome 19 determined by paired-end MNase-assisted H3 ChIP-seq. Black lines indicate DNA methylation in WT; red lines, DNA methylation in DKO ESCs. Within each plot, WT and DKO methylation was normalized in the same way and is quantitatively comparable. (*C*) Common nucleosomes whose boundaries change <5% between WT and DKO ESCs (>95% overlap between the bodies of the corresponding paired-end reads in WT and DKO cells). (*D*) Nucleosomes in WT cells whose boundaries were changed in DKO by >5% (<95% overlap). (*E*) Nucleosomes in WT cells whose boundaries were changed in DKO by >30% (<70% overlap). (*F*) All nucleosomes in DKO ESCs. (*G*) Fold enrichment of lost/gained nucleosomes at different genomic features. (*H*) Fold enrichment of common/gained/lost 5mC at genomic features.

We first tested our previous hypothesis that a 5mC/5hmC/5fC switch affects the nucleosome stability and their occupancy landscape (Teif et al. 2014) using MNase-assisted H3 ChIP-seq. We mapped regions with changing average nucleosome occupancy in DKO versus wild-type (WT) cells upon *Tet1/2* depletion within a 100-bp sliding window. This analysis identified 216,278 regions with increased and 22,365 regions with decreased nucleosome occupancy. We then calculated the average DNA methylation profiles in WT and DKO cells around the centers of these regions (Fig. 1A,B; also see Methods Online and Supplemental Fig. S1). The regions with decreased nucleosome occupancy were characterized by decreased DNA methylation, whereas those with increased occupancy showed increased methylation. To clarify the fine structure of DNA methylation inside and around nucleosomes, average methylation profiles around the centers of all nucleosomes were calculated. First, we considered nucleosomal DNA fragments that showed an overlap of at least 95% between WT and DKO cells (Fig. 1C). In this case, methylation was much higher inside nucleosomes, smoothly increasing from the middle of the nucleosome toward the ends and then dropping at the nucleosome ends and oscillating up to a distance of ∼1 kb from the nucleosome center with a period equal to the nucleosome repeat length (NRL). Second, this calculation was repeated for nucleosomes that shifted by >5% (Fig. 1D) or >30% (Fig. 1E). Methylation profiles were significantly changed around nucleosomes that shifted between WT and DKO ESCs, and we were able to track down methylation changes to the regions inside nucleosomes that undergo a shift >30% (Fig. 1E). Third, all nucleosomes in DKO cells were considered. In this case, the methylation profile inside the nucleosome was reversed compared with the WT profile (Fig. 1F). We obtained a similar picture, albeit without oscillations, when considering only nucleosomes inside CpG islands (Supplemental Fig. S2).

Next, we quantified changes of nucleosome occupancy at different genomic features. Although the majority of regions increased their nucleosome occupancy in DKO ESCs, a significant number of functional genomic elements (promoters, enhancers, CpG islands, regions marked by 5hmC in WT cells, 5hmC-to-5mC substitutions in DKO ESCs, and TAD boundaries) showed decreased nucleosome occupancy upon *Tet1/2* knockout (Fig. 1G; Supplemental Figs. S3, S4, S5A). Nucleosome loss was particularly pronounced for CpG islands and regions marked by 5fC and TET1 in WT cells. Regions marked by 5fC in WT ESCs (Song et al. 2013) were characterized by much stronger nucleosome loss in comparison with those marked by 5hmC or 5caC. This effect was also confirmed using another 5fC data set with single–base pair resolution (Xia et al. 2015), which showed a 2.52-fold enrichment of regions with decreased nucleosome occupancy and 0.25-fold depletion of regions with increased nucleosome occupancy at 5fC sites.

A Gene Ontology (GO) analysis of genomic regions that lost nucleosomes in DKO ESCs showed an enrichment for pluripotency-related processes (PluriNetWork [Som et al. 2010], *P* = 0.0087) and for DNA sequence motifs of EGR1 (*P* = 0.0016) (Supplemental Tables S1, S2). EGR1 is known to regulate hematopoietic differentiation (Nguyen et al. 1993). We found the expression of *Egr1* slightly increased in DKO cells (1.26-fold, *P* = 6.4 × 10^−4^) (Supplemental Table S3). This may suggest that *Tet1/2* depletion affects differentiation pathways in accordance with the hematopoietic differentiation defects observed in *Tet2*-deficient mice (Li et al. 2011). Regions that gained nucleosomes were enriched for binding motifs of the TATA box binding protein TBP (*P* = 0.037) (Supplemental Table S5), although no changes in *Tbp* expression were observed. In general, genes significantly up-regulated in DKO ESCs were enriched for the GO categories meiosis (*P* = 1.6 × 10^−5^), myosin (*P* = 6.4 × 10^−4^), differentiation (*P* = 0.0016), hematopoietic cell lineage (*P* = 7.3 × 10^−4^), and immunity (*P* = 0.0028). Up-regulated genes that gained nucleosomes at their promoters also followed this trend, with an additional enrichment for glycoproteins (*P* = 1.6 × 10^−4^) (Supplemental Tables S4–S6). Genes significantly down-regulated in DKO cells were not enriched with clusters of GO terms using the same criteria.

Next, we looked at the genome-wide statistics of methylome changes. Any gain of 5mC in DKO ESCs reflects methylated cytosine, whereas the observed loss of 5mC in DKO cells can be owing to the loss of either 5hmC or 5mC, because both marks are not distinguished by bisulfite sequencing (Huang et al. 2010). In line with the increase of average nucleosome occupancy, we also observed a global increase in DNA methylation; 9,739,847 CpGs changed their methylation level from <20% in WT to >50% in DKO cells. Figure 1H and Supplemental Figure S5B show how 5mC was redistributed in DKO relative to WT ESCs. Gained 5mC sites were less frequent in CpG islands in comparison to common and lost 5mC sites. Promoters tended to keep their methylation status, whereas enhancers displayed increased levels of changed methylation (both lost and gained 5mC). This may indicate extensive modulation of gene expression by changes of DNA methylation at enhancers.

### Common and lost CTCF sites have different CpG patterns

To study the effect of DNA methylation and nucleosome positioning on functional CTCF sites, we applied a stringent filter to analyze the CTCF ChIP-seq data. We considered only those CTCF sites that appeared in all technical and biological replicates for a given cell type (WT and DKO). Based on this criterion, 7232 CTCF sites were present in both cell types (“common” sites), and 3916 CTCF sites were lost in DKO ESCs compared with WT (“lost” sites; for example regions, see Supplemental Figs. S7–S15). Only 44 sites appeared in both DKO replicates and were not found in any WT replicate (“gained” sites; these were not further considered in the downstream analysis). Differences in CTCF expression between WT and DKO cells measured by RNA-seq were <10%, indicating that changes in binding do not simply reflect CTCF expression changes (Supplemental Table S3). Furthermore, our western blot data showed similar CTCF abundance at the protein level in WT and DKO cells (Supplemental Fig. S6).

**Figure 2.**
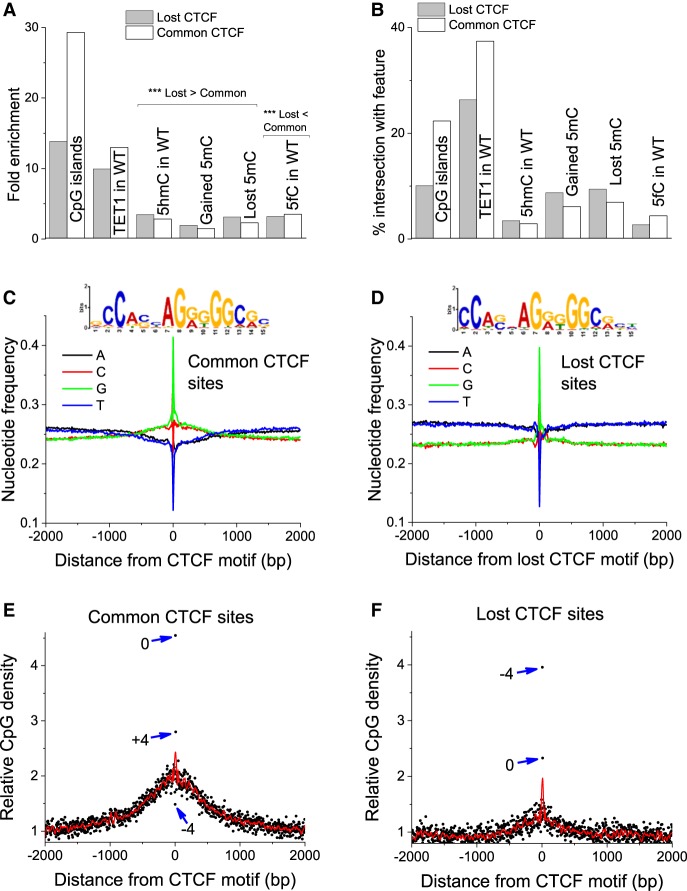
Loss of CTCF is associated with reduced GC content and CpG density. (*A*,*B*) Fold enrichment (*A*) and percentage overlap (*B*) of lost/common CTCF sites with different genomic features. CTCF sites are defined as 19-nucleotide motifs within the corresponding CTCF ChIP-seq peaks. (*C*,*D*) The nucleotide frequencies within ±2000 bp around CTCF motifs in common (*C*) and lost (*D*) peaks, as well as the corresponding consensus motifs. (*E*,*F*) CpG density around CTCF motifs in common and lost sites. Black dots correspond to individual CpG positions, red lines represent a spline interpolation of their density, and blue arrows indicate the outstanding CpGs inside the CTCF binding motif together with their coordinates with respect to the central peak of CpG density.

For the CTCF peaks defined above, we mapped the presence of the 19-bp CTCF binding motif and identified 18,000 common and 11,123 lost CTCF sites. On average, a given peak contained two to three copies of the CTCF motif. Figure 2, A and B, show the statistics of common and lost CTCF sites defined by DNA motifs. Common CTCF sites were twice more frequently detected inside CpG islands compared with lost CTCF sites. In contrast, the enrichment of common/lost CTCF sites with hydroxymethylated or differentially methylated sites showed the opposite tendency: Lost CTCF sites were significantly enriched at sites that changed their 5mC status. With respect to 5mC oxidation products, we found that lost CTCF sites were significantly more associated with 5hmC in WT ESCs than common sites and were significantly less associated with 5fC than common sites (Fig. 2A,B).

We then tested the hypothesis that common and lost CTCF sites have different probabilities to be methylated owing to different CpG content. Indeed, 52% of common CTCF motifs contained CpGs, whereas only 42% of lost CTCF sites contained CpGs. Thus, more than half of the lost CTCF sites did not contain CpGs and were therefore not directly affected by DNA methylation. Although common and lost CTCF sites were characterized by the same canonical CTCF motif, they had distinct differences. Lost sites, on average, showed a weaker match with the CTCF motif and had lower GC content in comparison to common sites (Fig. 2C,D). The CpG content of common and lost sites showed a similar pattern (Fig. 2E,F). Thus, common but not lost CTCF sites were surrounded by regions with higher GC content and enriched with CpGs, whereas lost sites had a decreased probability to contain CpGs inside the CTCF motif in comparison with common sites.

**Figure 3.**
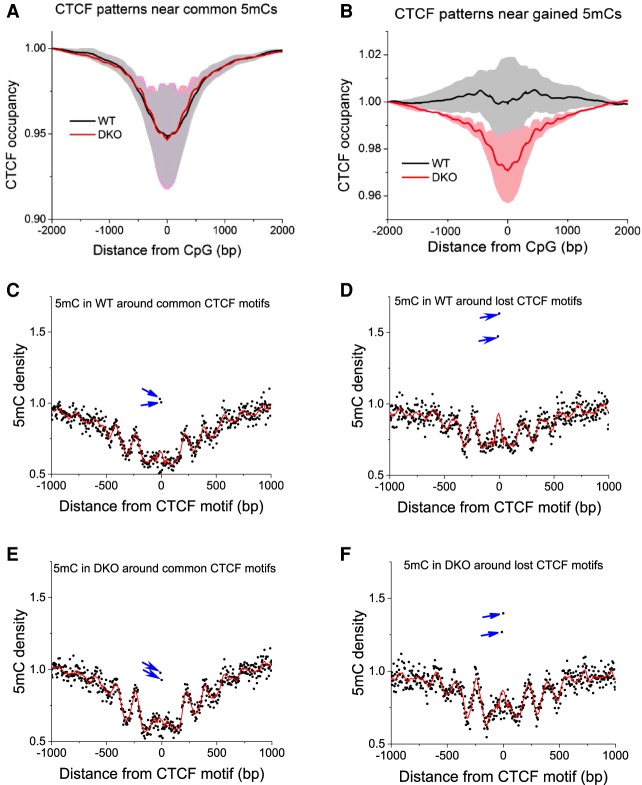
Genome-wide CTCF rearrangement happens preferentially at CpGs that gain methylation in DKO ESCs. (*A*) The average CTCF occupancy profiles around commonly methylated CpGs (methylation >0.8 both in WT and DKO cells; *N* = 10,505,682). (*B*) Depletion of CTCF occupancy around CpGs that gain methylation in DKO ESCs (<20% methylation in WT, >50% methylation in DKO cells; *N* = 9,739,847). CTCF profiles have been first calculated for individual replicate experiments and then averaged for all available replicates correspondingly for each cell type. Gray/light red shaded areas show the standard deviations of this averaging. (*C*–*F*) 5mC density around common and lost CTCF motifs in WT and DKO cells. Black dots correspond to individual CpG positions, red lines represent a spline interpolation of their density, and blue arrows indicate outstanding CpGs inside the CTCF binding motif.

We also performed an integrated analysis of DNA methylation and CTCF binding in WT and DKO ESCs. The average profiles of CTCF occupancy in the vicinity of commonly methylated CpGs did not change upon TET knockout (Fig. 3A). In contrast, CpGs that changed their methylation status were characterized by changes of CTCF occupancy. The most significant change of CTCF binding was observed for a class of CpGs changing their methylation status from low (average methylation <0.2) to intermediate and high (average methylation >0.5) (Fig. 3B). DNA methylation around common and lost CTCF motifs showed characteristic profiles with well-defined oscillations (Fig. 3C–F). The methylation level inside CTCF binding sites was reduced in common, but increased in lost, CTCF sites (Fig. 3C–F). This feature was characteristic for both WT and DKO 5mC profiles. Common and lost CTCF sites also showed different CpG patterns (Fig. 2), suggesting that some of the common and lost sites may have different modes of CTCF binding.

### CTCF binding is determined by DNA sequence, methylation, and nucleosome occupancy

**Figure 4.**
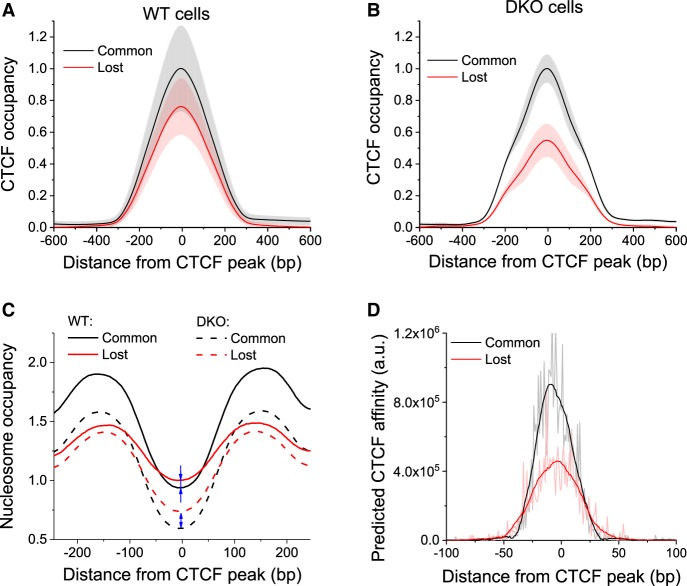
CTCF loss in DKO ESCs is predetermined by weaker DNA sequence affinities at a subset of lost sites. (*A*,*B*) Normalized average CTCF occupancy profiles around common and lost CTCF sites in WT (*A*) and DKO cells (*B*). Black line indicates common sites; red line, lost sites. Gray and light red shaded areas show the corresponding standard deviation. (*C*) Normalized average nucleosome occupancy profiles around common and lost CTCF sites in WT and DKO ESCs. Blue arrows show that nucleosome occupancy at lost sites was higher than at common sites both in WT and DKO cells, but in DKO ESCs, this difference becomes larger. (*D*) CTCF affinity predicted by the biophysical model from the DNA sequence for regions around common and lost CTCF sites was about twofold higher for common sites.

In several instances, changes in CTCF binding occurred at sites with differential methylation/nucleosome occupancy (Supplemental Figs. S7–S17). To assess this relation systematically, we predicted differential CTCF binding based on DNA sequence, changes of methylation, and nucleosome positioning. We calculated average CTCF occupancy profiles around common and lost sites for all replicate experiments (Supplemental Figs. S16, S18), averaged all replicates separately for each of the two cell types (WT and DKO), and normalized to equal CTCF occupancy at common sites (Fig. 4A,B; Supplemental Fig. S18). Lost sites were mostly present in DKO ESCs, and consistent with the concept of CTCF–nucleosome competition, the nucleosome occupancy at lost CTCF sites increased in DKO cells (Fig. 4C).

Further analysis showed that the predicted (based on DNA sequence) CTCF affinity of lost sites was lower than that of common sites (Fig. 4D) and quantitatively reproduced the experimental distribution in Figure 4B. Thus, it was possible to distinguish the subset of CTCF sites lost in DKO ESCs based on their weaker affinity for CTCF-DNA binding. Our comparison of different predictors of CTCF loss revealed that the strength of the CTCF binding motif was an equally good predictor as the change of nucleosome occupancy (AUC = 0.57 in both cases) (Supplemental Fig. S19A). In contrast, the level of DNA methylation could not be used to predict CTCF loss at individual sites. Consistent with the data in Figure 2, E and F, the best predictor of CTCF loss was the CpG density in regions of 1000 bp surrounding CTCF sites. CTCF binding was lost from sites surrounded by low CpG density and retained at sites with high CpG density (AUC = 0.65) (Supplemental Fig. S19A). These results support our model of the 5mC/5hmC/nucleosome switch (Supplemental Fig. S19B): Inside CpG islands, CTCF binding is mostly invariant, whereas outside of CpG islands, CTCF binding is determined by CTCF/nucleosome competition, which in turn is determined by DNA methylation through changes of nucleosome stability and location.

### DNA sequence features link CTCF binding and DNA methylation

**Figure 5.**
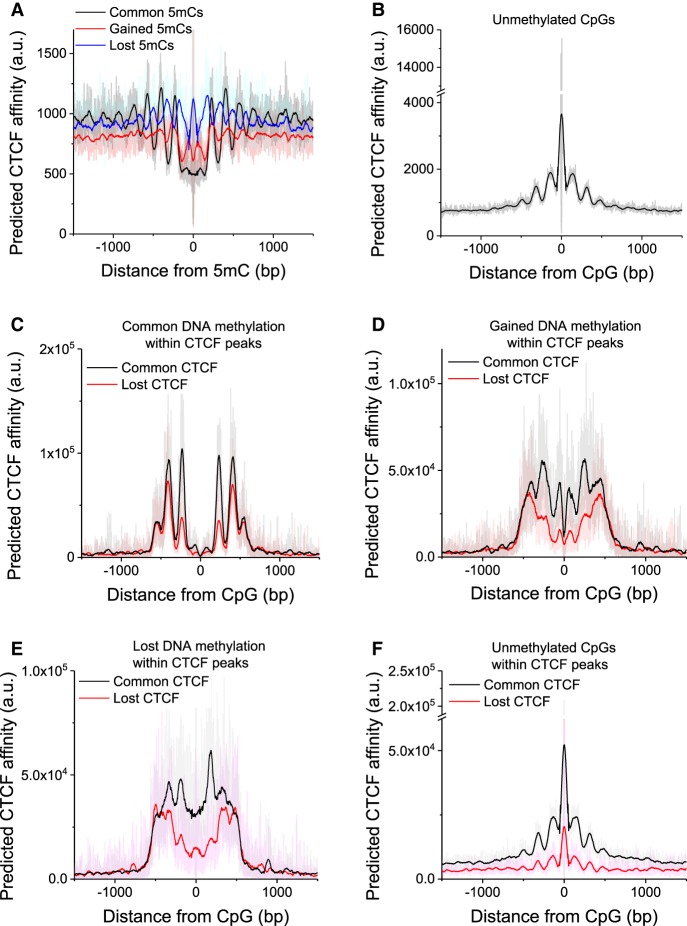
CTCF-DNA binding affinity predicted from the DNA sequence as a function of distance from CpGs. (*A,B*) Calculations performed for four classes of CpGs genome-wide. (*A*) CpGs that were commonly methylated in both cell states (methylation >0.8 both in WT and DKO cells; *N* = 10,439,081), that gained methylation (<0.2 in WT and >0.5 in DKO ESCs; *N* = 9,596,997), and that lost methylation (>0.5 in WT and <0.2 in DKO ESCs; *N* = 6,859,738). (*B*) Unmethylated CpGs (<0.2 in both WT and DKO; *N* = 15,316,892). (*C–F*) Calculations performed only for CpGs within CTCF ChIP-seq peaks in WT. (*C*) Common 5mC sites inside common (*N* = 25,740) and lost (*N* = 33,060) CTCF peaks. (*D*) Gained 5mC sites inside common (*N* = 37,702) and lost (*N* = 35,518) CTCF peaks. (*E*) Lost 5mC sites inside common (*N* = 35,632) and lost (*N* = 35,527) CTCF peaks. (*F*) Unmethylated CpGs inside common (*N* = 460,752) and lost (*N* = 179,288) CTCF peaks.

To further dissect the long-range effects of CpG content on CTCF binding, we analyzed the correlation of CTCF motifs and DNA methylation. Figure 5, A and B, shows average profiles of genome-wide predicted CTCF affinity as a function of the distance from CpGs, characterized by common, lost, and gained methylation (Fig. 5A), as well as for commonly unmethylated CpGs (Fig. 5B). The average sequence-determined CTCF energy landscapes were different for all four CpG categories. CpGs unmethylated both in WT and DKO cells were characterized by higher CTCF binding, whereas methylated CpGs showed decreased CTCF binding. We also observed that the CTCF energy profiles around gained and lost 5mC regions were in counter-phase. Lost 5mC sites were characterized by a peak of CTCF affinity at the center, whereas gained sites were characterized by a CTCF affinity drop. In all four cases, the CTCF energy landscape oscillated with a periodicity of 176 ± 3 bp (determined by the NRL in those regions, which was >10 bp smaller than the genome-wide NRL).

Further analysis revealed that commonly methylated/unmethylated CpGs were associated with very similar profiles for common and lost CTCF peaks, with some differences in CTCF affinity (Fig. 5C,F). In contrast, CpGs that gained/lost methylation displayed different shapes (Fig. 5D,E). These calculations were repeated for regions inside and outside of CpG islands, as well as inside and outside of promoters (Supplemental Figs. S20, S21), showing that the periodicity was mainly determined by the regions outside promoters and CpG islands. Furthermore, CTCF affinity peaks inside promoters and CpG islands were associated with peaks of local CpG density (Supplemental Figs. S20C, S21C). Thus, the connection between DNA methylation changes and CTCF loss appears to be dependent on the DNA sequence in a larger region surrounding CTCF sites.

### CTCF loss at functional elements near genes is linked to reduced gene expression

**Figure 6.**
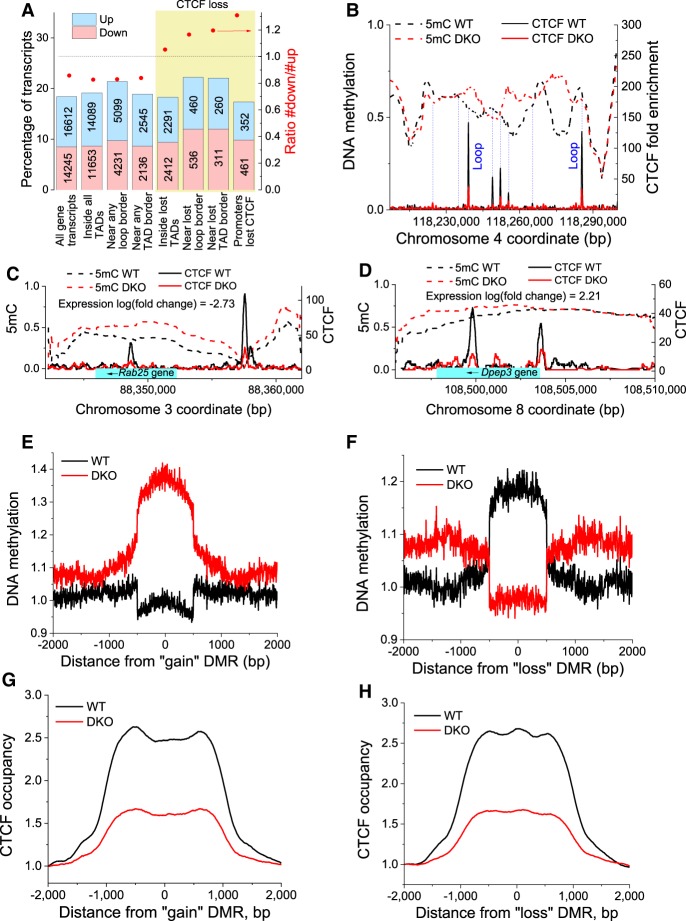
*Tet1/2* knockout changes DNA methylation profiles separated by CTCF and influences gene expression. (*A*) Changes of gene expression upon *Tet1/2*-dependent loss of CTCF from functional genomic regions. The bars show percentages of up- and down-regulated transcripts with respect to all transcripts overlapping with a given feature. The values on the bar indicate the corresponding numbers of transcripts in each category. Bars numbered *left* to *right*: (1) all transcripts genome-wide; (2) transcripts inside all TADs; (3) transcripts within 10 kb from any loop boundary; (4) transcripts within 10 kb from any TAD boundary; (5) transcripts located within TADs that lost a boundary (a boundary was called lost if there was at least one lost CTCF site within 10 kb from the boundary); (6) transcripts within 10 kb from any lost loop boundary based on the same criterion for the boundary loss; (7) transcripts within 10 kb from any lost TAD boundary based on the criterion for the boundary loss; and (8) transcripts that lost CTCF from their promoters. The yellow area indicates features that lost CTCF. The red points correspond to the ratio of the numbers of down- versus up-regulated transcripts indicated on the *right* axis. (*B*–*D*) Example genomic regions showing the DNA methylation pattern smoothed with a 500-bp sliding window as it changes between WT and DKO cells. Thick dashed lines show average 5mC level per CpG, and solid lines show CTCF occupancy in WT (black) and DKO (red) ESCs. Thin blue dashed lines indicate peaks of CTCF occupancy. Some of these coincide with chromatin loop borders reported by Bonev et al. (2017) (indicated on the figure). Light blue rectangle shows the gene body. The arrow indicates direction of transcription. Gene expression changes are indicated in the figure. (*E*,*F*) DNA methylation profiles in WT (black) and DKO (red) cells around centers of 1000-bp regions that were characterized by increased (“gain”) or decreased methylation (“loss”) in DKO ESCs. (*G*,*H*) Average CTCF occupancy profiles around “gain” and “loss” DMRs, showing that “gain” DMRs tended to be flanked by CTCF sites. The same effect was observed for “loss” DMRs but was less evident because of a fraction of CTCF sites located in the middle of “loss” DMRs.

Next, we analyzed the effect of differential CTCF binding on gene expression (Fig. 6). Transcripts were annotated based on their expression changes and location with respect to individual CTCF sites, boundaries of topologically associated domains (TADs), and chromatin loops reported in WT ESCs (Bonev et al. 2017). Genome-wide, we observed a tendency of more up-regulated than down-regulated transcripts in DKO cells (see the leftmost bar in Fig. 6A). The same trend was observed inside and outside loops or TADs, both close to the boundaries of loops and TADs and far away from them, as long as CTCF loss was not taken into account (see the first four bars in Fig. 6A). However, inside TADs that lost boundaries, this relation was reversed (more transcripts were down-regulated than up-regulated), which was even more pronounced in the vicinity of these lost boundaries. Finally, transcripts that contained lost CTCF sites in their promoters showed an even stronger tendency for down-regulation (see the rightmost bar in Fig. 6A). This effect was statistically significant in all gene classes characterized by CTCF loss described above (χ^2^ test, *P* < 6.6 × 10^−5^). Thus, CTCF loss was correlated with a down-regulation of gene expression within the corresponding domain demarcated by CTCF in WT cells. This effect includes whole domains that lost boundaries and has a strong distance-dependent component. It was more pronounced close to the lost CTCF sites compared with regions within the same TAD but located distantly from lost CTCF sites (Fig. 6A).

An explanation for the observed distance-dependent effect of CTCF loss on gene expression could be changes of DNA methylation as a function of the distance from the lost CTCF site. As shown in Figure 6, B through D, and Supplemental Fig. S22, DNA methylation averaged with a sliding window of 500 bp yields smooth landscapes for WT and DKO ESCs that partly coincide and partly deviate from each other. These methylation profiles were demarcated by CTCF sites in two ways. First, some CTCF sites were located in the summits of high-methylation peaks or the bottoms of low-methylation valleys. Similar behavior has been reported previously, suggesting that CTCF can prime neighboring regions for demethylation (Stadler et al. 2011). Second, some CTCF sites appeared to act as boundaries for methylation spreading. The loss of CTCF from these sites turns them into “bifurcation points,” when on one or both sides of the CTCF boundary the average 5mC profiles start diverging between WT and DKO cells.

To study the latter effect genome-wide, we analyzed differentially methylated genomic regions (DMRs) using the DMRcaller R package (Catoni et al. 2018) with a scanning window of 1000 bp for DMRs that lost (Fig. 6E) and gained (Fig. 6F) methylation in DKO cells. This analysis revealed that both “loss” and “gain” DMRs were preferentially demarcated by CTCF (Fig. 6G,H; Supplemental Figs. S23, S24), which corresponds to CTCF acting as a bifurcation point in our examples in Figure 6, B through D, and Supplemental Figure S22. In addition, “loss” DMRs had increased occurrence of CTCF sites in the center of the DMR, which corresponds to CTCF positioned at the peak summits and valley bottoms of the methylation landscapes in Figure 6, B through D, and Supplemental Figure S22. However, DNA sequence motif analysis did not reveal CTCF as the top binding candidate for the regions near DMR boundaries, suggesting that additional TFs might be involved (Supplemental Table S7).

The asymmetry of DNA methylation profiles surrounding CTCF sites noted in Figure 6 would suggest that the CTCF distribution around methylated CpGs would also be asymmetric. To find out whether such asymmetry is hard-wired in the DNA sequence genome-wide, we computed the predicted CTCF binding affinity around different classes of CpGs based on their methylation status in WT and DKO cells and then performed *k*-means clustering of CTCF profiles of these regions (Supplemental Fig. S25). This analysis confirmed that clusters with asymmetric CTCF affinity distribution were characteristic for common or gained 5mC sites but not unmethylated CpGs and not for random regions (Supplemental Fig. S25). Thus, CTCF sites act as bookmarks for the demethylation process, appearing both at the methylation peak centers and at the boundaries, thereby separating regions of differentially methylated DNA.

## Discussion

**Figure 7.**
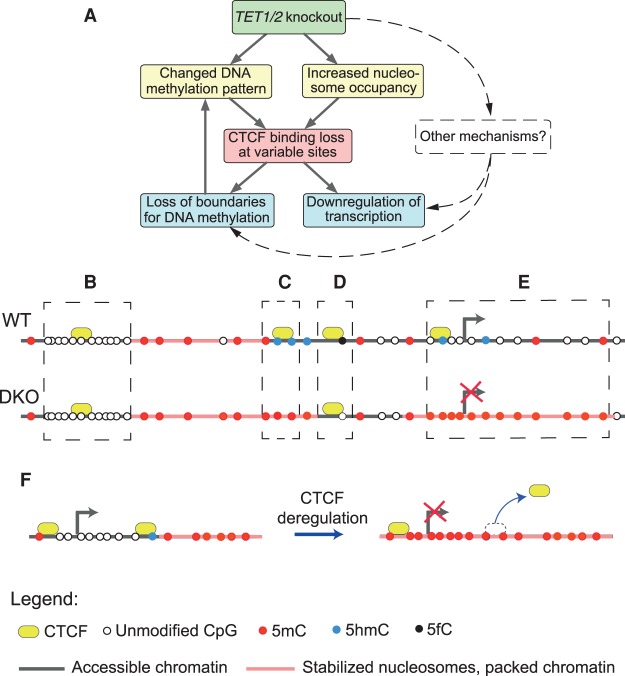
A scheme of different regimes of CTCF sensitivity to DNA modifications. (*A*) Simplified scheme of the possible causality of events: *Tet1/2* knockout leads to the changed DNA methylation pattern and increased nucleosome occupancy. These lead to CTCF binding loss at variable sites. As a result, methylation spreads to larger areas, and neighboring genes are down-regulated. (*B*) Common CTCF sites were significantly enriched at CpG islands where DNA was unmethylated in both cell types, and CTCF binding was mostly determined by the DNA sequence. (*C*) CTCF sites marked by 5hmC in WT were predisposed for loss of CTCF binding in DKO cells, which could be accompanied by a 5hmC/5mC switch and the loss of 5hmC. (*D*) Regions near 5fC sites were more enriched for common than for lost CTCF sites. (*E*) CTCF loss at promoters and in the vicinity of genes may lead to the spreading of DNA methylation into neighboring regions as a function of the distance from the CTCF site. Genes inside such regions tend to become down-regulated in DKO ESCs. (*F*) In some cases, methylation of a single CpG inside a CTCF binding site may lead to CTCF removal, or vice versa, and results in the loss of the boundary between methylation microdomains. This process may induce a subsequent change of transcription, as shown for an example genomic region in Supplemental Figure S27.

Mouse ESCs that lack TET1/2 enzymes display a genome-wide loss of 5hmC and a severe deregulation of the 5mC landscape (Dawlaty et al. 2013). In the present study, we link CTCF binding, DNA (de)methylation, and nucleosome occupancy by comparing WT ESCs with DKO ESCs that lack *Tet1/2*. The resulting cascade of downstream events can be summarized as follows (see also Fig. 7A; Supplemental Fig. S26): In DKO cells, nucleosome occupancy became reduced at sites that lost 5mC and increased at sites that gained 5mC. The latter effect was about 10 times more frequent. Sites losing nucleosomes were enriched at regulatory regions related to developmental and differentiation-related pathways, most likely leading to additional impairment of gene regulation. Our analysis suggested that the 5mC/nucleosome linkage is strongest within the nucleosomal DNA (Fig. 1) and uncovered distinct effects of 5mC, 5hmC, and 5fC at nucleosomes. Nucleosome loss was pronounced for regions marked by 5fC in WT cells. This may be related to different effects of 5fC and 5hmC on nucleosome stability. We reported previously that 5fC is associated with well-positioned nucleosomes, whereas 5hmC is associated with labile MNase-sensitive nucleosomes (Teif et al. 2014). A strong nucleosome-stabilizing effect of 5fC was explained recently by the formation of noncovalent bonds between formylated DNA and histones (Raiber et al. 2018). Different DNA cytosine modifications are known to modulate physically the rigidity and geometry of the double helix and, thus, nucleosome stability (Raiber et al. 2015; Dans et al. 2016; Ngo et al. 2016). In addition, the effects observed here might also be modulated by interactions with chromatin proteins that can selectively recognize unmodified and modified CpGs (Zhu et al. 2016).

Significant loss of CTCF binding was observed in DKO ESCs. In the minority of cases, this coincided with a 5mC/5hmC/5fC switch inside the CTCF binding motif, where CTCF loss was associated with deregulation of cytosine modifications. However, in the majority of cases, CTCF loss was associated with a nucleosome/5mC switch in the neighboring area rather than a methylation change inside the CTCF motif itself. In this context, CTCF loss could affect DNA methylation by removing some of the foci of methylation domains and some of the boundaries preventing spreading of methylation to the neighboring areas (Fig. 6). Thus, the interplay of DNA methylation and CTCF redistribution was not limited to an anticorrelation of CTCF binding and DNA methylation, as has been reported in previous studies (Stadler et al. 2011; Feldmann et al. 2013; Teif et al. 2014; Maurano et al. 2015). Rather, it included several conclusions that are summarized in Figure 7B: (1) CpG islands displayed a reduced frequency of CTCF loss from its binding sites; (2) the presence of 5fC, 5hmC, and 5mC modifications strongly affected the nucleosome/CTCF competition; and (3) a spreading of DNA methylation/demethylation and associated deregulation of neighboring genes was observed upon loss of CTCF binding at boundary elements.

Common and lost CTCF sites contained the same consensus motif, which was characterized by different methylation patterns in WT and DKO cells, consistent with previous observations (Hashimoto et al. 2017). Unlike common CTCF sites, lost sites did not have a pronounced CpG in the motif's center (Fig. 2). On the other hand, lost CTCF sites had a higher probability of containing methylated CpGs both in WT and DKO cells (Fig. 3). Furthermore, there were distinct patterns beyond the core CTCF motif: Common CTCF sites were embedded in larger regions with high GC and CpG content (presumably CpG islands). This is consistent with our previous reports showing that in different mouse and human cell types, CTCF binding perturbations indicated that CTCF is preferentially retained inside CpG islands (Teif et al. 2014; Pavlaki et al. 2018). We have also reported previously that DNA methylation canyons, which often overlap with CpG islands (Jeong et al. 2014), tagged with activating chromatin marks are less prone to hypermethylation upon *Tet1/2* loss (Wiehle et al. 2016). This may account for the particular preservation of CTCF binding in these regions with high GC and CpG content. It should also be noted that a previous study concluded that CTCF sensitivity to methylation is associated with CpG islands in the human HCT116 cell line (Maurano et al. 2015). Accordingly, further studies are needed to assess whether these effects are cell-type–specific.

Our quantitative model showed that the affinity of the CTCF motif and nucleosome occupancy were both comparable predictors of CTCF loss upon *Tet1/2* depletion. However, the best predictor was the DNA sequence of a larger ∼1-kb region encompassing the CTCF binding site (Fig. 4). This novel finding may explain why previous models for differential CTCF binding based on the modification/occupancy of the core CTCF motif had limited predictive power. We also showed that the average profile of DNA-encoded CTCF affinity oscillates with the NRL periodicity as a function of the distance from a CpG. The latter result has important implications, suggesting that regular arrays of nucleosomes around CTCF sites may be at least partially encoded in the DNA sequence and are not just a consequence of the boundary conditions on the statistical nucleosome density distribution (Fig. 5). DNA sequence–encoded nucleosome periodicity near CTCF binding sites was proposed in our previous work (Beshnova et al. 2014), and the sequence-encoded oscillations described in Figure 5 confirm this hypothesis. The concept that some TF binding sites are premarked in ESCs for later binding during development by DNA hydroxymethylation has also been put forward in a recent study (Kim et al. 2018). How exactly this premarking is achieved is not known. Our study suggests that the DNA sequence not only defines the genomic binding pattern for a given time point but also at least partially determines the future dynamics of differential DNA methylation and TF binding.

Several findings obtained here point to a role of CTCF sites as bifurcation points where the smooth differential DNA methylation profile changes its pattern upstream of and downstream from CTCF in regions that comprise several kilobases (Fig. 6). To our knowledge, such asymmetry has not been noticed before for genomic regions at this scale. A potentially related effect is the asymmetry of hemi-methylated CpGs flanking CTCF binding sites (Xu and Corces 2018). Another recent study considered averaged DNA methylation profiles around all TAD or intra-TAD boundaries and showed that DNA methylation levels smoothly decay as a function of the distance to the boundary (Matthews and Waxman 2018). Thus, CTCF might act as a DNA methylation insulator element. This happens at a relatively small percentage of DMR boundaries that contain CTCF sites, in line with previous reports that some CTCF sites do not act as a boundary for methylation spreading (Dickson et al. 2010). It is noted that CTCF can also act as a barrier between chromatin states that are characterized, for example, by H3K27me3 and H2AK5ac marks (Cuddapah et al. 2009).

Our observation that CTCF can set bifurcation points for the DNA methylation landscape might also explain the recently reported differential silencing of variably methylated repeat elements bordered by CTCF (Kazachenka et al. 2018). As depicted in Figure 7F, extended genomic regions of changed DNA methylation upstream of or downstream from lost CTCF “insulator” sites might lead to the deregulation of neighboring genes (for specific examples of such genes, see Supplemental Figs. S27–S31). Although there was a genome-wide preference for up-regulation of gene expression in DKO versus WT cells, this trend was reversed inside TADs that lost boundaries (which had more down-regulated than up-regulated genes). It was more pronounced for genes close to the lost boundaries of TADs and chromatin loops, as well as genes that lost CTCF from their promoters (Fig. 6A). These results align well with two recent knockout studies (Nora et al. 2017; Rao et al. 2017). In one of these studies, the removal of the CTCF interaction partner cohesin was linked to down-regulation of nearby superenhancers (Rao et al. 2017). The second work reported large gene expression changes after CTCF knockout, although it did not link them mechanistically to CTCF removal (Nora et al. 2017). Thus, the 5mC/5hmC/5fC/nucleosome/CTCF switch dissected here provides a new mechanistic model on how CTCF binding is modulated and how it could affect gene regulation.

## Methods

### ESC culture

WT and *Tet1/2*-deficient (DKO) mouse ES cell lines isolated from WT and *Tet1/Tet2* double-mutant mice with a mixed 129 and C57BL/6 background (Dawlaty et al. 2013) were maintained in regular ESC medium as detailed in the Supplemental Methods. For experiments, cells were trypsinized and preplated on gelatin-coated dishes three times to remove feeders.

CTCF ChIP-seq was performed as described previously (Wiehle and Breiling 2016) and sequenced in 50-bp single-read mode on an Illumina HiSeq 2000 device, as detailed in Supplemental Methods.

### MNase-assisted H3 ChIP-seq

Cells were cross-linked with 1% methanol-free formaldehyde for 10 min. After quenching with glycine, cells were washed three times with PBS. The cell pellet was treated with 40 U MNase for 5 min at 37°C and then stopped with 10× Covaris buffer (Covaris), and chromatin was sheared for 15 min with the Covaris S2 device (burst 200; cycle 20%; intensity 8). Immunoprecipitation was performed for approximately 5 × 10^6^ cells with anti-H3 antibody (Abcam ab1791, lot GR103864-1). Then chromatin was treated with RNase A and Proteinase K. Purified DNA was cloned into Illumina libraries with the NEBNext ultra library preparation kit (NEB). Paired-end reads were sequenced using Illumina HiSeq 2000.

RNA-seq was performed using total RNA extracted using a DNA-Free RNA kit (Zymo Research) as detailed in the Supplemental Methods. Libraries were prepared from RNA of WT and DKO ESCs using the TruSeq RNA sample preparation kit v2 (Illumina), clustered on cBot (Illumina) using TruSeq SR Cluster Kit v3 and sequenced by single-read 50-bp mode on a HiSeq 2000 v3 platform according to Illumina's instructions. RNA-seq analysis was performed in Genomatix (Genomatix GmbH) as detailed in the Supplemental Methods.

### Bisulfite sequencing

DNA fragmentation was performed using the Covaris S2 AFA system as detailed in the Supplemental Methods. End repair of fragmented DNA was performed using the paired-end DNA sample prep kit (Illumina). The ligation of the adaptors was performed using the Illumina early access methylation adaptor oligo kit (Illumina). The size selection of the adaptor-ligated fragments was performed using the E-Gel electrophoresis system (Invitrogen) and a size select 2% precast agarose gel (Invitrogen) as detailed in the Supplemental Methods. For the bisulfite treatment, we used the EZ-DNA methylation kit (Zymo Research) as detailed in the Supplemental Methods. The libraries were subsequently amplified, using the fast start high fidelity PCR system (Roche) with buffer 2 and the Illuminas PE1.1 and PE2.1 amplification primers as detailed in the Supplemental Methods. Base calling was performed with Illumina CASAVA 1.8.1 software, followed by trimming and quality filtering by Shore 0.6.2 (Ossowski et al. 2008) and downstream processing by BSmap 2.0 (Xi and Li 2009). The computation of methylation ratios was performed with the script methratio.py (part of the BSmap package). In the downstream analysis, commonly methylated CpGs were defined as those with methylation ≥0.8 in both cell states; gained 5mC, with methylation <0.2 in WT and >0.5 in DKO cells; lost 5mC, with methylation >0.5 in WT and <0.2 in DKO ESCs; and commonly unmethylated, with methylation <0.2 in both states.

### Western blot

WT and DKO ESCs were lysed and fractionated as described previously (Wysocka et al. 2001). The chromatin fraction was resolved by standard SDS-PAGE, and membranes were immunostained using antibodies against CTCF (61311, Active Motif) and H3 (Abcam ab1791, lot GR232149).

### Nucleosome occupancy analysis

Paired-end H3 ChIP-seq reads were mapped to the mouse genome mm9 using Bowtie (Langmead et al. 2009) allowing up to two mismatches and only unique alignments. This resulted in total 343 and 316 million mapped mononucleosome fragments correspondingly in WT and DKO cells (including two biological replicates both for WT cells and for DKO cells). Reads were then processed using the NucTools pipeline (Vainshtein et al. 2017) as detailed in the Supplemental Methods.

### CTCF ChIP-seq analysis

After mapping reads using Bowtie (Langmead et al. 2009), allowing up to two mismatches and only unique alignments, we obtained 58 million mapped reads in WT (two biological replicates named WT4 and WT6 and an additional technical replicate in WT6) and 33 million reads in DKO ESCs (two replicates named DKO26 and DKO51). CTCF peaks were determined with MACS (Zhang et al. 2008) using default parameters as detailed in the Supplemental Methods. Lost sites were defined as appearing in all replicates in WT while not appearing in any of DKO replicates. Gained sites were defined as appearing in all replicates in DKO and not appearing in any of WT replicates. Locations of CTCF motifs within CTCF peaks were determined by scanning for the CTCF motif from JASPAR (Mathelier et al. 2016) using RSAT with default parameters (Castro-Mondragon et al. 2017).

### CTCF affinity calculation

For the CTCF binding affinity calculation, we implemented a MATLAB version of the TRAP algorithm described elsewhere (Roider et al. 2007), as detailed in the Supplemental Methods. The choice of the TRAP constant R_0_ = 10^9^ and the energy mismatch scale λ = 1.5 were the same as in our previous work (Teif et al. 2014), with the CTCF PWM taken from the JASPAR database (Mathelier et al. 2016). Clustering of the unsmoothed CTCF affinity profiles was performed using ClusterMapsBuilder in NucTools (Vainshtein et al. 2017) on a sample of 200,000 available affinity profiles for each case based on the values of the logarithm of the predicted affinity. For the background clustering control, a set of 50,000 random genomic region samples was generated using BEDTools (Quinlan and Hall 2010). Receiver-operator curves were calculated using Origin 2018 (OriginLab) as detailed in the Supplemental Methods.

GO analysis was performed with Enrichr (Kuleshov et al. 2016) and DAVID v 6.7 (Huang da et al. 2009) as detailed in the Supplemental Methods. Adjusted Benjamini *P*-values were used throughout the manuscript unless stated otherwise in the text.

### DMR calling

To determine DMRs, we used the R/Bioconductor package DMRcaller (Catoni et al. 2018) with a sliding window of 1000 bp, calling all regions where the average methylation level in a given window deviated between WT and DKO cells by >10%.

### External data sets

The 5hmC map in WT ESCs was taken from GSM882244 (Yu et al. 2012). 5fC maps in WT ESCs were taken from GSE41545 (used in our Fig. 1; Song et al. 2013) and from GSE66144 (Xia et al. 2015). 5caC was taken from Shen et al. (2013). TET1 binding sites in WT ESCs were taken from GSM611192 (Williams et al. 2011). All these data sets were aligned by their investigators to the mm9 mouse genome. Hi-C data determining the boundaries of TADs and promoter–enhancer loops were taken from Bonev et al. (2017). These were initially aligned to GRCm38 (mm10), and we have converted them to mm9 using the liftOver tool of the UCSC Genome Browser in order to use mm9 for all manipulations in this manuscript. Realigning the reads to mm10 would not significantly affect the conclusions because the coordinates of most genomic regions could be uniquely converted between these two genome assemblies.

## Data access

The raw sequencing data generated in this study have been submitted to the NCBI Gene Expression Omnibus (GEO; https://www.ncbi.nlm.nih.gov/geo/) under accession numbers GSE110460 (bisulfite sequencing) and GSE114599 (ChIP-seq and RNA-seq). Scripts developed in this study have been uploaded as Supplemental Code and are also available at https://github.com/TeifLab/TFaffinity.

## Supplementary Material

Supplemental Material
