# Supplementary material for: DNA (de)methylation in embryonic stem cells controls CTCF-dependent chromatin boundaries
Source: Genome Res. 2019 May;29(5):750–61. doi: 10.1101/gr.239707.118 (PMC6499307; doi:10.1101/gr.239707.118)
Supplement: Supplemental Material [file supp_29_5_750__index.html]

DNA (de)methylation in embryonic stem cells controls CTCF-dependent chromatin boundaries — Supplemental Material 

# DNA (de)methylation in embryonic stem cells controls CTCF-dependent chromatin boundaries

## Supplemental Material

- Supplemental\_Materials.pdf
- Supplemental\_Table\_ST3.zip
- Supplemental\_Code.zip
