# Supplementary material for: DNA (de)methylation in embryonic stem cells controls CTCF-dependent chromatin boundaries
Source: Genome Res. 2019 May;29(5):750–61. doi: 10.1101/gr.239707.118 (PMC6499307; doi:10.1101/gr.239707.118)
Supplement: Supplemental Material [file supp_gr.239707.118_Supplemental_Materials.pdf]

Supplemental Materials

DNA (de)methylation in embryonic stem cells controls CTCF-dependent chromatin boundaries

Laura Wiehle[1,6,a], Graeme J. Thorn[2,4,a], Günter Raddatz[1], Christopher T. Clarkson[2], Karsten Rippe[3], Frank Lyko[1], Achim Breiling[1,5,*] and Vladimir B. Teif[2,*]

[1] Division of Epigenetics, DKFZ-ZMBH Alliance, German Cancer Research Center (DKFZ), 69120 Heidelberg, Germany.

[2] School of Biological Sciences, University of Essex, Wivenhoe Park, Colchester, CO4 3SQ, UK.

[3] Division of Chromatin Networks, German Cancer Research Center (DKFZ) & Bioquant, Heidelberg, 69120, Germany.

[4] Current address: Barts Cancer Institute, Queen Mary University of London, Charterhouse Square, London, EC1M 6BQ, UK.

[5] Current address: EMBO Press, Meyerhofstrasse 1, 69117 Heidelberg, Germany.

[6] Current address: Institute of Human Genetics, Ulm University and Ulm University Medical Center, 89081 Ulm, Germany.

[a] These authors contributed equally

* Correspondence should be addressed to Vladimir B. Teif (vteif@essex.ac.uk) or Achim Breiling (achim.breiling@embo.org)

Supplemental Methods

ESC culture. WT and *Tet1/2*-deficient (DKO) mouse ES cell lines isolated from WT and Tet1/Tet2 double-mutant mice with a mixed 129 and C57BL/6 background (Dawlaty et al., 2013) were maintained in regular ESC medium (KnockOut DMEM supplemented with 15% fetal calf serum, 1x non-essential amino acids, 100 U/ml penicillin and 100 µg/ml streptomycin, 2 mM L-glutamine, 0.0008% β-mercaptoethanol and 1000 U/ml leukemia inhibiting factor) at 37 °C and 7% CO_2 on inactivated MEF feeders seeded on gelatin (0.2%) coated dishes. For experiments, cells were trypsinized and pre-plated on gelatin coated dishes three times to remove feeders.

ChIP-seq. ESCs were resuspended in medium, fixated for 10 min at room temperature and lysed as described (Wiehle and Breiling 2016). After nuclei lysis, samples were sonicated 4 × 5 min with 30 s on/off intervals (Bioruptor, Diagenode). SDS content was reduced by dilution as described (Wiehle and Breiling 2016). Chromatin from 6×10^6 cells was immunoprecipitated overnight at 4°C using 5 µg of antibody against CTCF (#61311, Active Motif). Further processing occurred according to instructions of the ChIP-IT High Sensitivity kit (Active Motif). Immunoprecipitated DNA was eluted from Protein A/G agarose beads and purified using the IPure kit (Diagenode). Libraries for ChIP-sequencing were generated from 10 ng of DNA using the NEBNext ChIP-Seq Library Prep Master Mix Set (NEB) according to the manufacturer´s instructions. Final libraries were pooled to equimolar amounts and sequenced in 50 bp single-read mode on an Illumina HiSeq 2000 device.

MNase-assisted ChIP-seq. Cells were cross-linked with 1% methanol-free formaldehyde for 10 min. After quenching with glycine, cells were washed three times with PBS. The cell pellet was treated with 40 U MNase for 5 min at 37°C, then stopped with 10x Covaris buffer (Covaris Ltd) and chromatin was sheared for 15 min with the Covaris S2 device (burst 200; cycle 20%; intensity 8). Immunoprecipitation was performed for ~5×10^6 cells with anti-H3 antibody (Abcam #ab1791, Lot: GR103864-1). Then chromatin was treated with RNase A and proteinase K. Purified DNA was cloned into Illumina libraries with the NEBNext Ultra library preparation kit (NEB).

Nucleic acids isolation. Genomic DNA was isolated by lysing freshly prepared cell pellets in pre-lysis buffer (10 mM Tris-HCl (pH 8), 5 mM EDTA, 100 mM NaCl, 1.1% (v/v) SDS, 0.1 mg/ml Proteinase K and 0.04 mg/ml RNAse A) at 37°C overnight. On the next day proteins were precipitated by addition of 5 M NaCl and the DNA was isolated by isopropanol precipitation. Total RNA was isolated from freshly harvested cells using TRIzol (Invitrogen).

RNA-seq. For transcriptome sequencing total RNA was subjected to DNA digestion using a DNA-Free RNA Kit (Zymo Research). Libraries were prepared from RNA of WT and DKO ESCs using the TruSeq RNA Sample Preparation Kit v2 (Illumina). After quality control libraries were pooled, clustered on the cBot (Illumina) using the TruSeq SR Cluster Kit v3 and sequenced by single-read 50 bp mode on a HiSeq 2000 v3 platform (Illumina) according to Illumina´s instructions. Library preparation for bisulfite sequencing was performed using the TruSeq DNA PCR-Free LT Library Preparation Kit with modifications. Briefly, 2 µg of genomic DNA were fragmented to 150-200 bp using a Covaris ultrasonicator (Covaris, Inc), end-repaired, purified using 1.6x sample purification beads and 3´-adenylated. TruSeq LT adapters were ligated to the DNA fragments followed by bisulfite treatment using the EpiTect Bisulfite Kit (Qiagen) according to instructions of the Illumina WGBS for Methylation Analysis Guide (Part # 15021861 Rev. B). Fragment libraries were enriched with 8 cycles of PCR using KAPA HiFi Uracil+ DNA Polymerase with customized primers (forward: 5'-AATGATACGGCGACCACCGA-3', reverse: 5'-CAAGCAGAAGACGGCATACGAGAT-3') and an annealing temperature of 69 °C according to the settings for paired-end libraries in the technical datasheet (KAPA HiFi HotStart Uracil+ Ready Mix, KR0413 - version 1.12, peqlab). Amplified libraries were purified with 1x Agencourt AMPure XP beads (Beckman Coulter Inc), pooled, clustered on the cBot (Illumina) and sequenced in paired-end mode on a HiSeq2000 platform according to standard Illumina protocols.

Bisulfite sequencing. 5 µg of high molecular weight genomic DNA were used for fragmentation using the Covaris S2 AFA System in a total volume of 100 µl. Fragmentation-run parameters: Duty cycle 10%; Intensity: 5; Cycles/burst: 200; Time: 3 min; number of cycles: 3, resulting in a total fragmentation-time of 180 s. Fragmentation was confirmed with a 2100 Bioanalyzer (Agilent Technologies) using a DNA1000 chip. The fragmented DNAs were concentrated to a final volume of 75 µl using a DNA Speed Vac. End repair of fragmented DNA was carried out in a total volume of 100 µl using the Paired End DNA Sample Prep Kit (Illumina) as recommended by the manufacturer. For the ligation of the adaptors, the Illumina Early Access Methylation Adaptor Oligo Kit and the Paired End DNA Sample Prep Kit (Illumina) were used, as recommended by the manufacturer. For the size selection of the adaptor-ligated fragments, we used the E-Gel Electrophoresis System (Invitrogen) and a Size Select 2% precast agarose gel (Invitrogen). Each fragmented DNA was loaded on two lanes of the E-gel. Electrophoresis was carried out using the “Size Select” program for 16 min. According to the standard loaded (50 bp DNA Ladder, Invitrogen), 240 bp fragments were extracted from the gel, pooled, and directly transferred to bisulfite treatment without further purification. For the bisulfite treatment we used the EZ-DNA Methylation Kit (Zymo Research) as recommended by the manufacturer with the

exception of a modified thermal profile for the bisulfite conversion reaction. The conversion was carried out in a thermal cycler using the following thermal profile: 95°C for 15 s, 50°C for 1 h, repeat from step 1, 15×, 4°C for at least 10 min. The libraries were subsequently amplified, using the Fast Start High Fidelity PCR System (Roche) with buffer 2, and Illuminas PE1.1 and PE2.1 amplification primers. PCR thermal profile: 95°C for 2 min, 95°C for 30 s, 65°C for 20 s, 72°C for 30 s, then repeat from step 2, 11×, 72°C for 7min, hold at 4°C. PCR reactions were purified on PCR purification columns (MinElute, Qiagen) and eluted in 20 µl elution buffer (Qiagen). Base calling was performed with Illumina Casava 1.8.1 software, followed by trimming and quality filtering by Shore 0.6.2, and downstream processing by BSmap 2.0 (Xi and Li 2009). The computation of methylation ratios was done with the script methratio.py (part of the BSmap package). In the downstream analysis commonly methylated CpGs were defined as those with methylation ≥0.8 in both cell states; gained 5mC – with methylation <0.2 in WT and >0.5 in DKO cells; lost 5mC – with methylation >0.5 in WT and <0.2 in DKO ESCs; commonly unmethylated – with methylation <0.2 in both states.

Western Blot. WT and DKO ESCs were lysed and fractionated as described previously (Wysocka et al. 2001). The chromatin fraction was resolved by standard SDS-PAGE and membranes were immunostained using antibodies against CTCF (#61311, Active Motif) and H3 (Abcam ab1791, Lot: GR232149).

MNase-assisted H3 ChIP-seq analysis. Paired-end reads were mapped to the mouse genome mm9 using Bowtie (Langmead et al. 2009) allowing up to two mismatches and only unique alignments. This resulted in total 343 and 316 million mapped mono-nucleosome fragments correspondingly in WT and DKO cells (including two biological replicates named WT4 and WT6 for WT cells and DKO26 and DKO51 for DKO cells). Reads were then processed using the NucTools pipeline (Vainshtein et al. 2017) to calculate genome-wide occupancies and average aggregate profiles. The datasets of regions that lost and gained nucleosomes were calculated using NucTools with a sliding window of 100 bp, a nucleosome stability threshold 0.5, and the relative occupancy change threshold 0.99, where the relative occupancy change O_{diff} is defined as

$$O_{diff} = 2 \times (<O_{DKO}> - <O_{WT}>) / (<O_{DKO}> + <O_{WT}>)$$

Here $<O_{DKO}>$ is the replicate-averaged occupancy in DKO cells, and $<O_{WT}>$ is the replicate-averaged occupancy WT, using two replicates for each of DKO and WT ESCs. The enrichment of regions that lost/gained nucleosomes in different genomic features was calculated as the ratio N_{real}/N_{random}, where N_{real} is the number of regions that intersect

between a given dataset of nucleosome regions with a given genomic feature, and N_{random} is the number of regions intersected between the “random” dataset with a given genomic feature. The “random” dataset was produced by the BedTools shuffleBed command to match the number and size distribution of the corresponding real dataset with regions that lost/gained nucleosomes.

CTCF ChIP-seq analysis. We performed three replicate CTCF ChIP-seq experiments in WT and two replicate experiments in DKO cells. This resulted in 58 million mapped single-end reads in WT (2 biological replicates named WT4 and WT6 and two technical replicates in WT6) and 33 million mapped single-end reads in DKO ESCs (2 replicates named DKO26 and DKO51). CTCF peaks were determined with MACS (Zhang et al. 2008) using default parameters. The following number of peaks were detected: 34,900, 16,669 and 41,253 peaks in WT cells (the latter two numbers correspond to technical replicates WT6 cell line), and 12,970 and 15,939 peaks in two biological replicates in DKO cells. Common sites were defined as peaks appearing in all replicates in both cell conditions. Lost sites were defined as appearing in all replicates in WT while not appearing in any of DKO replicates. Gained sites were defined as appearing in all replicates in DKO and not appearing in any of WT replicates. Precise positioning of CTCF binding sites within each category of CTCF peaks was done by scanning for the CTCF motif from JASPAR (Mathelier et al. 2016) using RSAT with default parameters (Castro-Mondragon et al. 2017).

RNA-seq analysis. Differential RNA-seq analysis was performed in Genomatix (Genomatix GmbH) using the ElDorado transcript database. For the statistical tests with respect to expression changes near lost CTCF sites, we have based our analysis on transcripts whose expression satisfied log(fold change)>|0.5|, as illustrated on the violin plots below. In panel A we consider transcripts of all genes. When we consider transcripts with log(fold change)>|0.5|, there are more upregulated (16,612 in the red rectangle) than downregulated (14,245 in the blue rectangle) transcripts. On the contrary, in panel B violin plot is calculated for genes near lost loop borders and genes whose promoters lost CTCF, in which case there are less upregulated (352 in the red rectangle) than downregulated (451 in the blue rectangle) transcripts. Considering the distribution of these two groups of genes between two categories (upregulated and downregulated), the exact Fisher test gives the $p < 1.2 \times 10^{-9}$, proving the statistical significance of this result. The same considerations have been applied for all groups of genes shown in Figure 6A. When we consider gene expression changes near TAD borders, loop borders and promoters that lost CTCF, these are significantly different from genome-wide distribution, in favour of downregulation ($p < 6.6 \times 10^{-5}$).

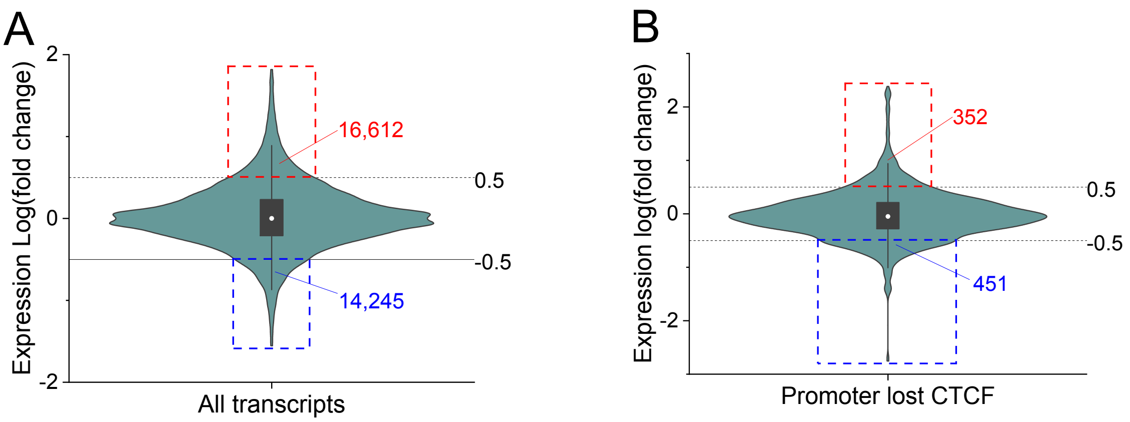

CTCF affinity calculation. For the CTCF binding affinity calculation, we implemented a MATLAB version of the TRAP algorithm described elsewhere (Roider et al. 2007). MATLAB scripts have been made available at https://github.com/TeifLab/TFaffinity. The choice of the TRAP constant $R_0 = 10^9$ and the energy mismatch scale $\lambda = 1.5$ were the same as in our previous work (Teif et al. 2014), with the CTCF PWM taken from the JASPAR database (Mathelier et al. 2016). Averaged profiles were calculated over all available sequences for that particular region type, and then shifted by half the width of the CTCF PWM so that the profiles were aligned relative to the CpG site or the center of CTCF ChIP-seq peak. Plotted average affinity profiles were smoothed using Savitzky-Golay filter of order 2 with a 100-bp window size using Origin 2018 (OriginLab). Clustering of the unsmoothed CTCF affinity profiles was performed using ClusterMapsBuilder in NucTools (Vainshtein et al. 2017), on a sample of 200,000 available affinity profiles for each case based on the values of the logarithm of the predicted affinity. For the background clustering control, a set of 50,000 random genomic region sample was generated using BEDTools (Quinlan 2014).

ROC analysis. Receiver-Operator Curves were calculated using Origin 2018 (OriginLab) based on the following input data. For predicting CTCF binding purely from the DNA sequence, CTCF affinity calculated as described above was compared with the experimental CTCF site classification into common and lost passed on MACS peak calling as detailed above. For predicting CTCF binding from the nucleosome occupancy, the experimental nucleosome occupancy averaged between two replicates in 100-bp windows around CTCF binding sites was used instead of CTCF affinity. For predicting CTCF binding from the CpG density the CpG density was averaged in 1000-bp windows around CTCF sites and used as input.

Gene Ontology analysis. Enrichment of pathways and TF binding in genomic regions that gained or lost nucleosomes was quantified with Enrichr (Kuleshov et al. 2016) using default

parameters. Functional annotation of differentially expressed genes was performed using DAVID v 6.7 using default settings (Huang da et al. 2009).

DMR calling. To determine differentially methylated regions we used the R/Bioconductor package DMRcaller (Catoni et al. 2018) with a sliding window of 1000bp calling all regions where the average methylation level in a given window deviated between WT and DKO cells by more than 10%.

External datasets. 5hmC map in WT ESCs was taken from GSM882244 (Yu et al. 2012). 5fC maps in WT ESCs were taken from GSE41545 (Song et al. 2013) (used in our Figure 1) and from GSE66144 (Xia et al. 2015). 5caC was taken from (Shen et al. 2013). TET1 binding sites in WT ESCs were taken from GSM611192 (Williams et al. 2011). Hi-C data determining the boundaries of topologically associated domains (TADs) and promoter-enhancer loops were taken from (Bonev et al. 2017).

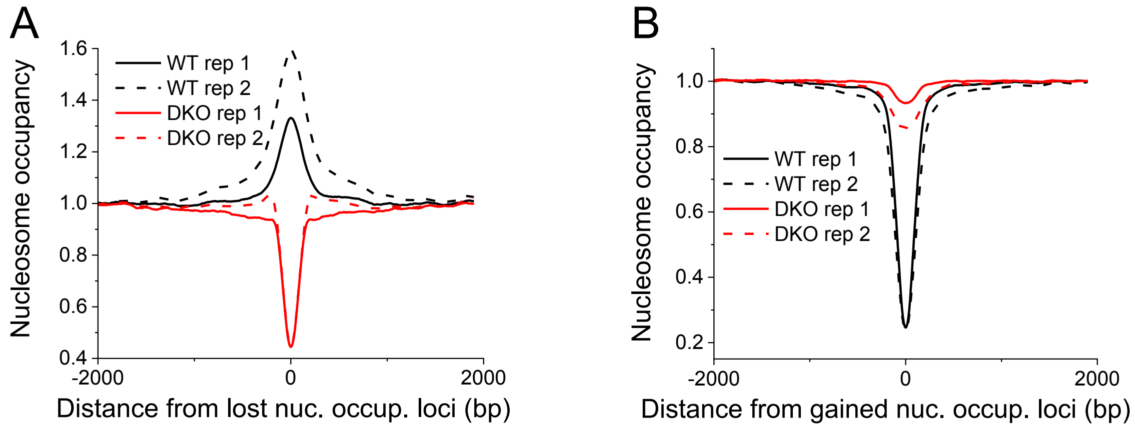

Figure S1. Average nucleosome occupancy profiles around genomic regions for which nucleosome occupancy decreased (denoted as "lost" nucleosomes) (A) or increased (denoted as "gained" nucleosomes) (B) in DKO cells. The NucTools algorithm with 100-bp sliding window was used.

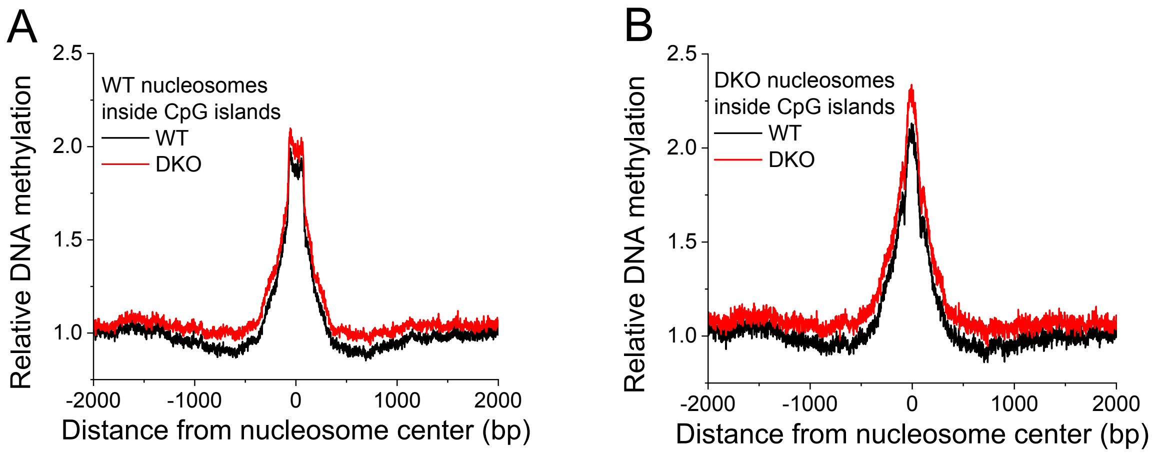

Figure S2. CpG islands are characterised by relatively conserved profiles of DNA methylation on the nucleosomes. Relative DNA methylation is plotted as a function of the distance from the centers of nucleosomes on chromosome 19 determined by paired-end MNase-assisted H3 ChIP-seq in WT (A) and DKO cells (B). Black lines – DNA methylation in WT. Red lines – DNA methylation in DKO. Within each plot the WT and DKO methylation levels are normalised in the same way and quantitatively comparable.

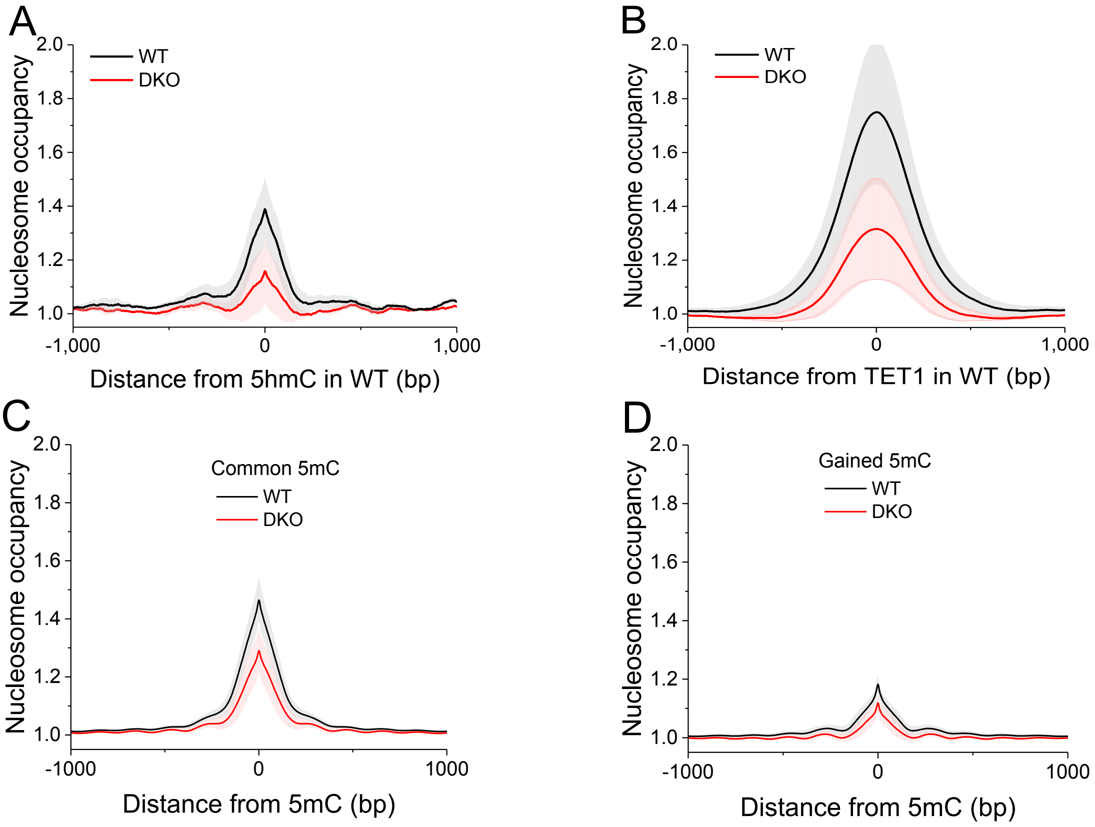

Figure S3. Average profiles of nucleosome occupancy in WT (black line) and DKO (red line) around 5hmC sites (A), TET1 bound in WT ESCs (B), common 5mCs (C) and gained 5mCs (D). The grey and pink areas show standard deviations.

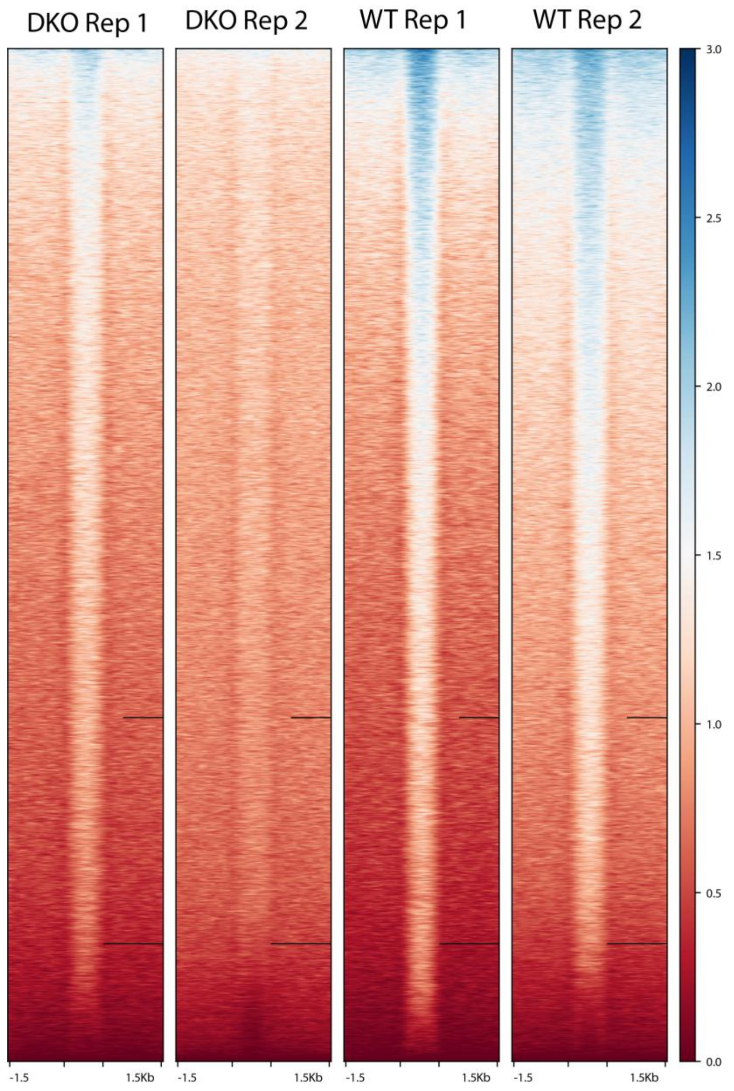

Figure S4. Heatmaps of nucleosome occupancy in DKO (two replicates, left) and WT (two replicates, right) around regions bound by TET1 in WT ESCs. Average profiles shown in Figure S3B are based on the same regions.

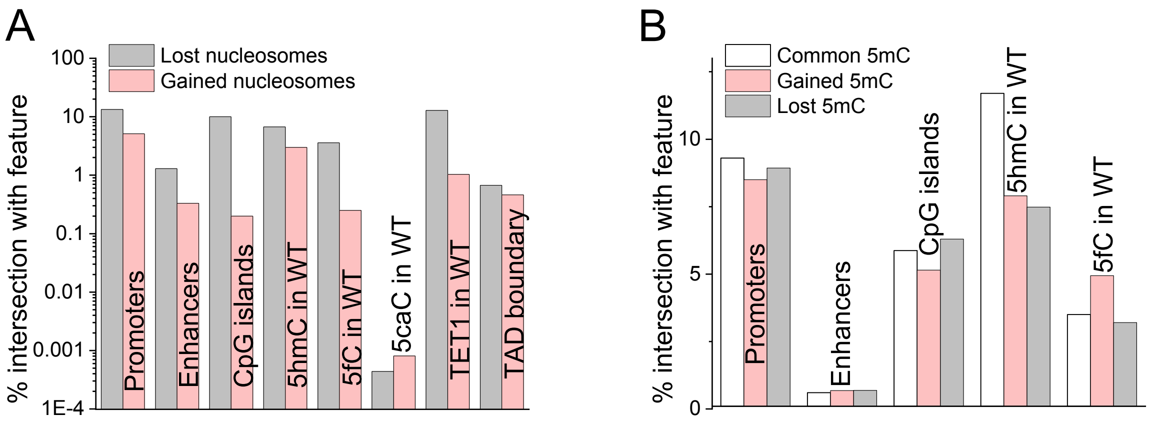

Figure S5. A) Percent overlap of lost (grey) and gained nucleosomes (pink) with different genomic features indicated on the figure. B) Percent overlap of common (white), gained (pink) and lost 5mCs (grey) with different genomic features indicated on the figure.

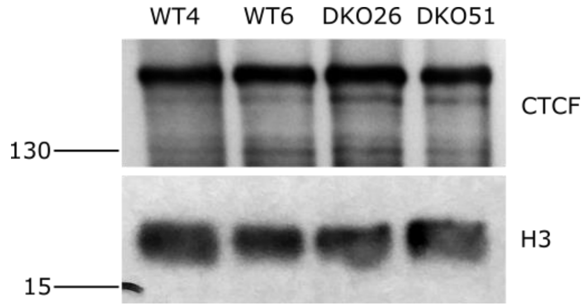

Figure S6. Western blot in WT and DKO cells with anti-CTCF and anti-H3 antibody.

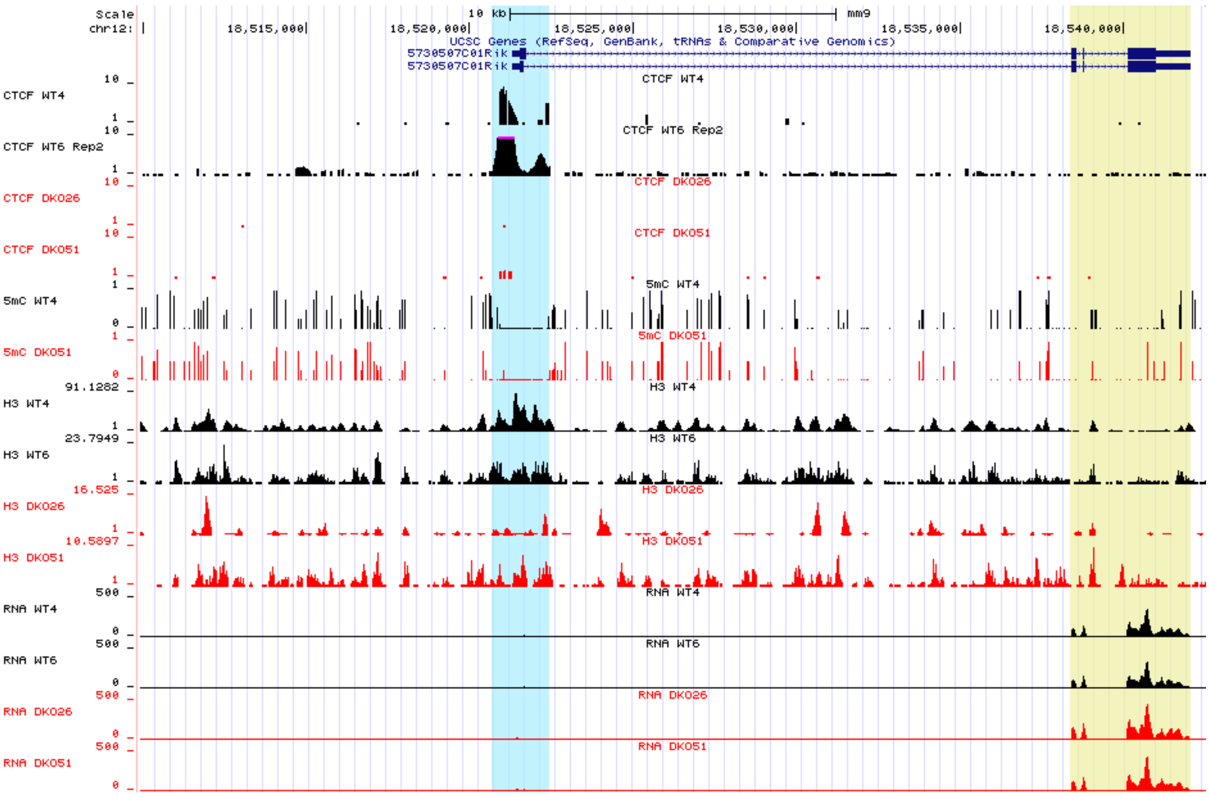

Figure S7. Example region on chromosome 12 showing tracks of raw signals of CTCF ChIP-seq (in wild-type cells WT4 and WT6 and in TET knockout cells DKO26 and DKO51), as well as whole genome bisulfite sequencing and RNA-seq in WT4 and DKO51 cells. Datasets corresponding to wildtype cells are in black and those corresponding to DKO cells are in red colour. Highlighted rectangles show regions of pronounced epigenome changes.

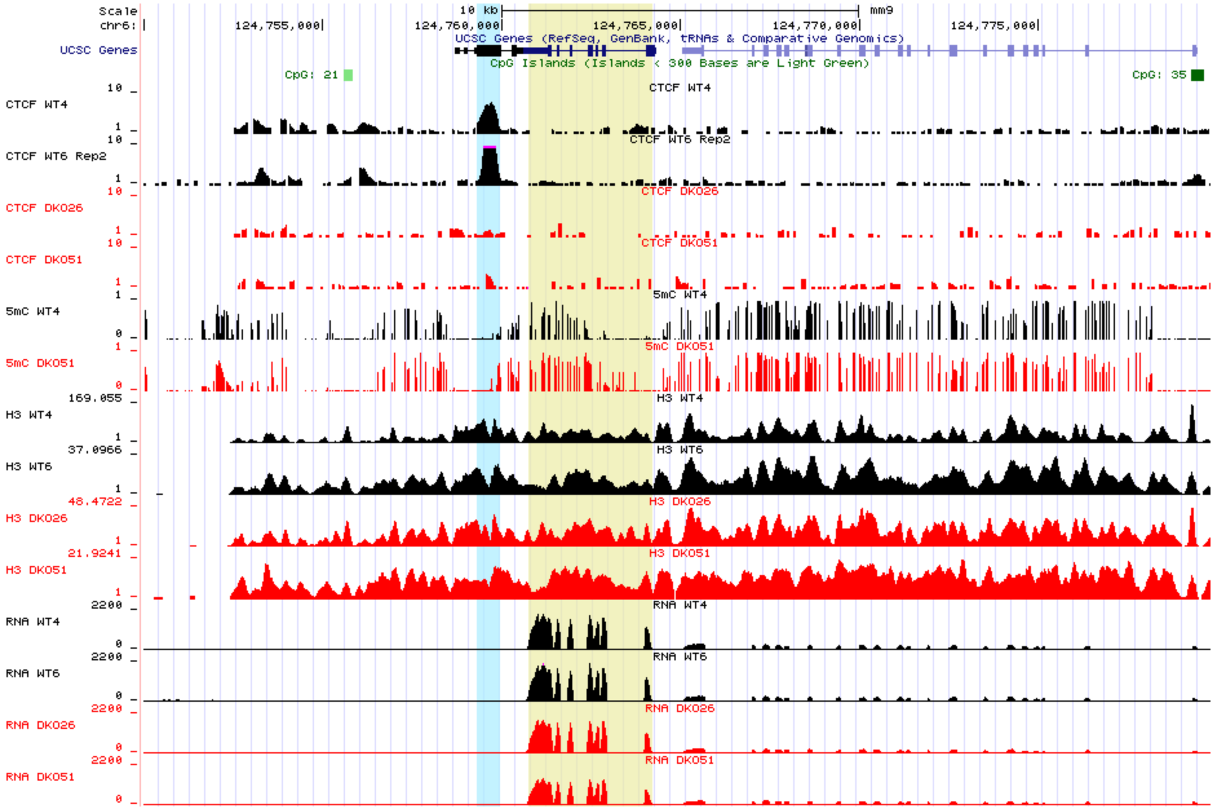

Figure S8. Example region on chromosome 6 showing tracks of raw signals of CTCF ChIP-seq (in wild-type cells WT4 and WT6 and in TET knockout cells DKO26 and DKO51) and whole genome bisulfite sequencing, as well as whole genome bisulfite sequencing and RNA-seq in WT4 and DKO51 cells. Datasets corresponding to wildtype cells are in black and those corresponding to DKO cells are in red colour. Highlighted rectangles show regions of pronounced epigenome changes.

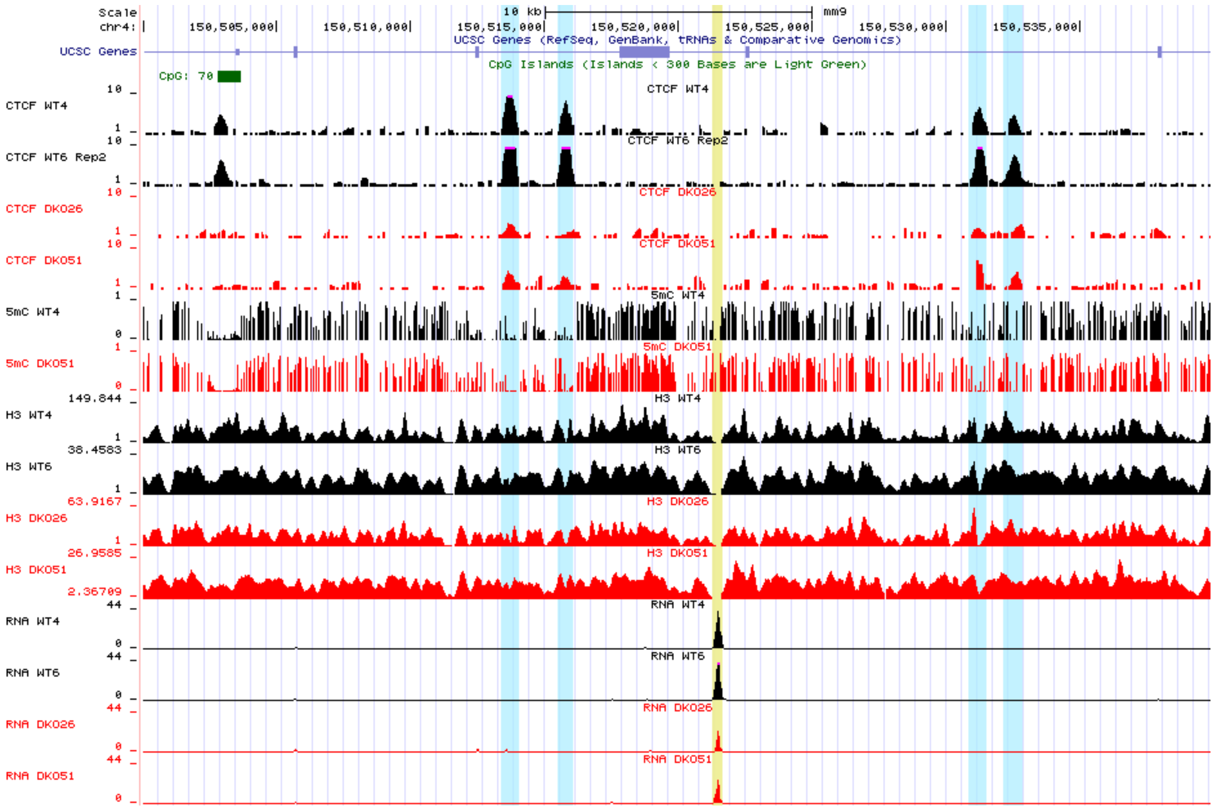

Figure S9. Example region on chromosome 4 showing tracks of raw signals of CTCF ChIP-seq (in wild-type cells WT4 and WT6 and in TET knockout cells DKO26 and DKO51), as well as whole genome bisulfite sequencing and RNA-seq in WT4 and DKO51 cells. Datasets corresponding to wildtype cells are in black and those corresponding to DKO cells are in red colour. Highlighted rectangles show regions of pronounced epigenome changes.

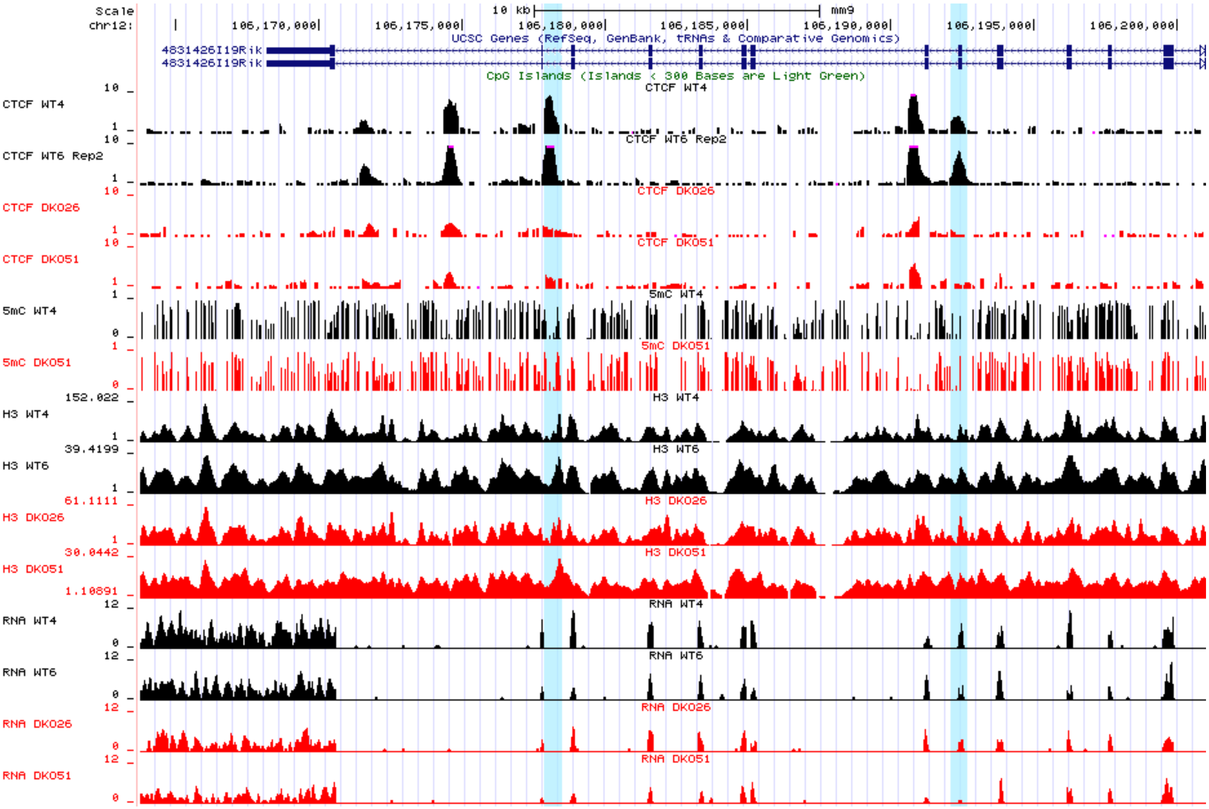

Figure S10. Example region on chromosome 12 showing tracks of raw signals of CTCF ChIP-seq (in wild-type cells WT4 and WT6 and in TET knockout cells DKO26 and DKO51), as well as whole genome bisulfite sequencing and RNA-seq in WT4 and DKO51 cells. Datasets corresponding to wildtype cells are in black and those corresponding to DKO cells are in red colour. Highlighted rectangles show regions of pronounced epigenome changes.

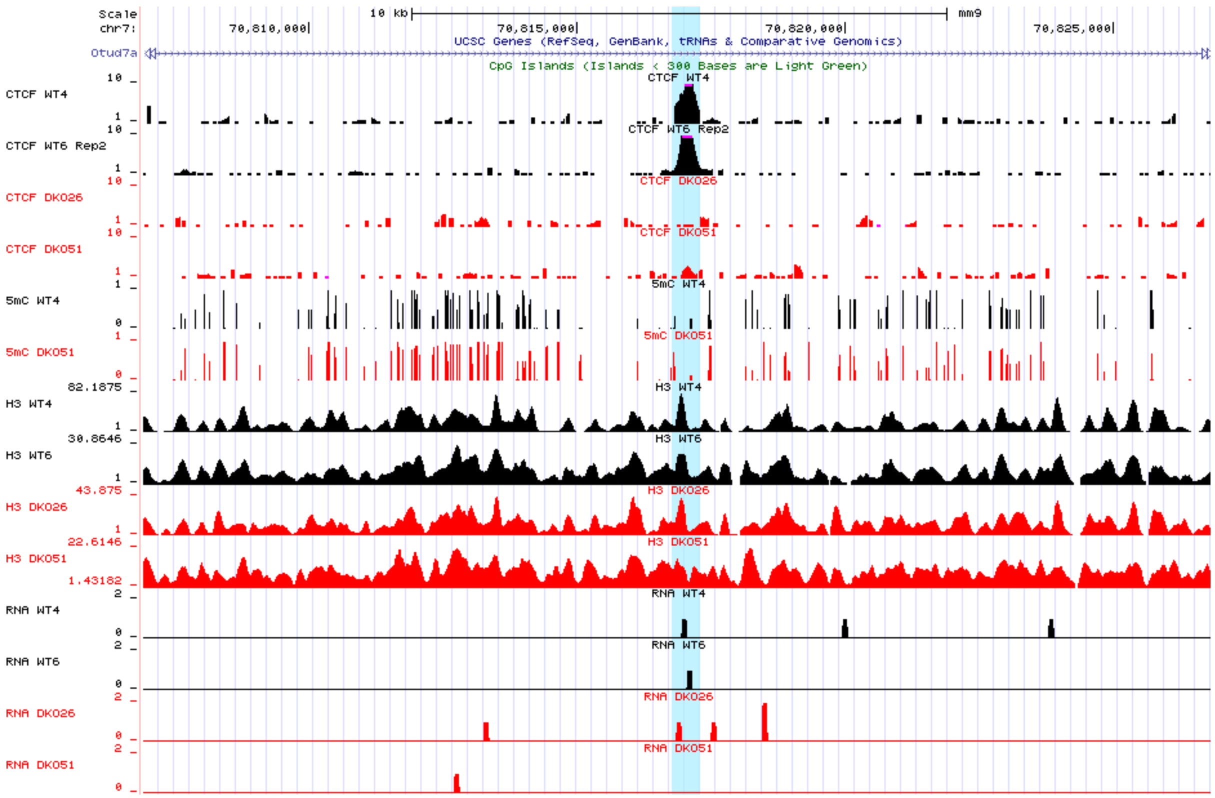

Figure S11. Example region on chromosome 7 showing tracks of raw signals of CTCF ChIP-seq (in wild-type cells WT4 and WT6 and in TET knockout cells DKO26 and DKO51), as well as whole genome bisulfite sequencing and RNA-seq in WT4 and DKO51 cells. Datasets corresponding to wildtype cells are in black and those corresponding to DKO cells are in red colour. Highlighted rectangles show regions of pronounced epigenome changes.

A

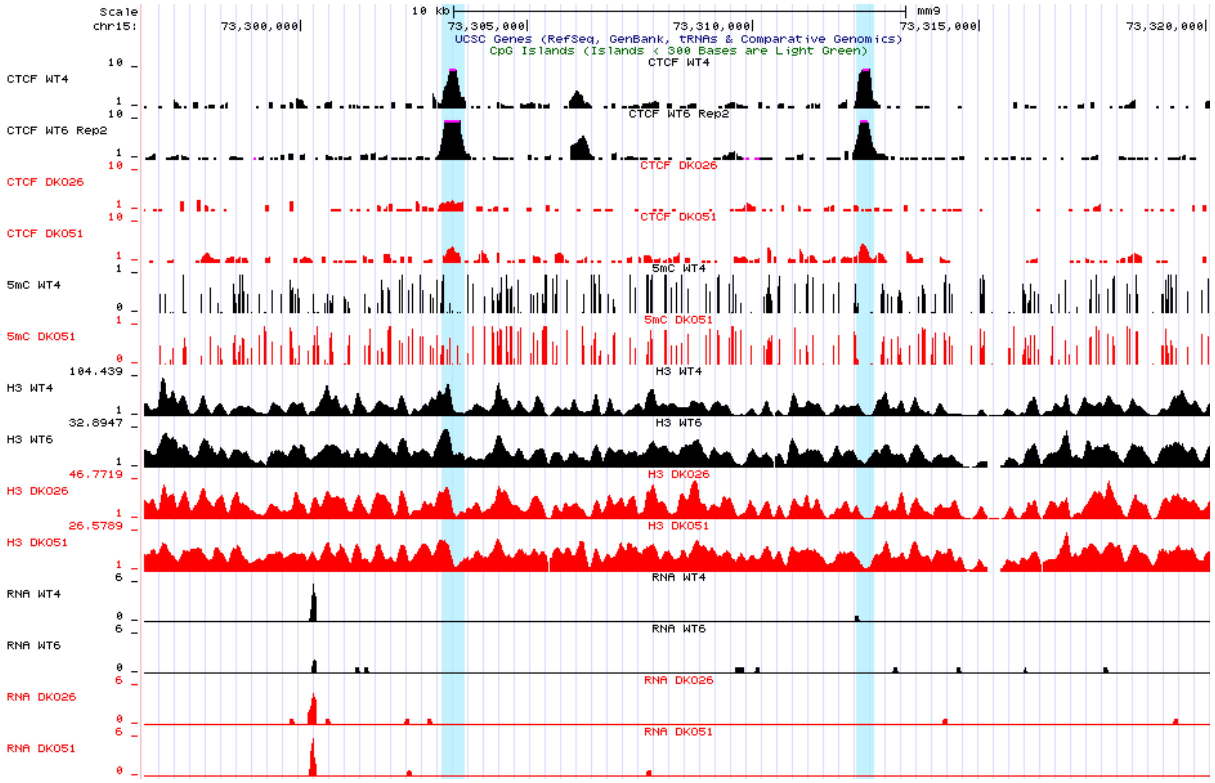

B

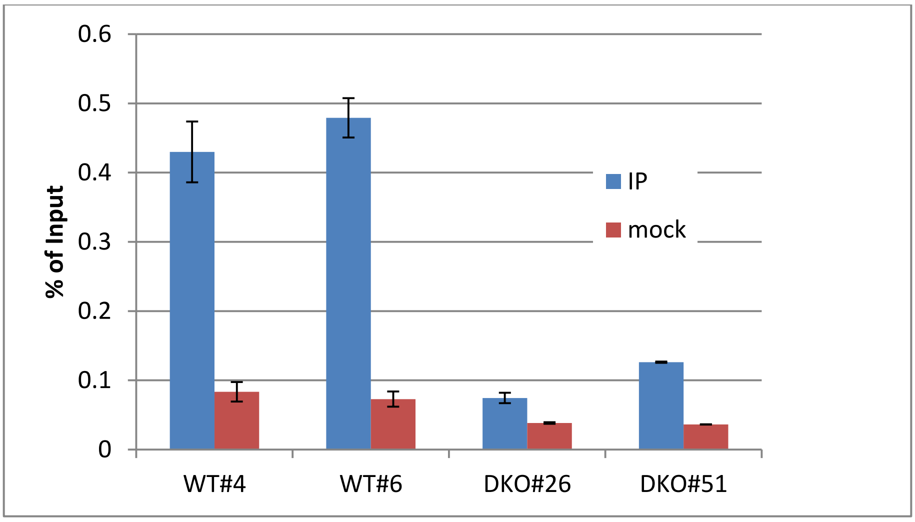

Figure S12. (A) Example region on chromosome 15 showing tracks of raw signals of CTCF and H3 ChIP-seq (in wild-type cells WT4 and WT6 and in TET knockout cells DKO26 and DKO51) and whole genome bisulfite sequencing and RNA-seq in WT4 and DKO51 cells. Highlighted rectangles show regions of pronounced CTCF binding changes. (B) qPCR validation of CTCF binding loss in DKO for the peak shown above at position chr15:73305892-73306519.

A

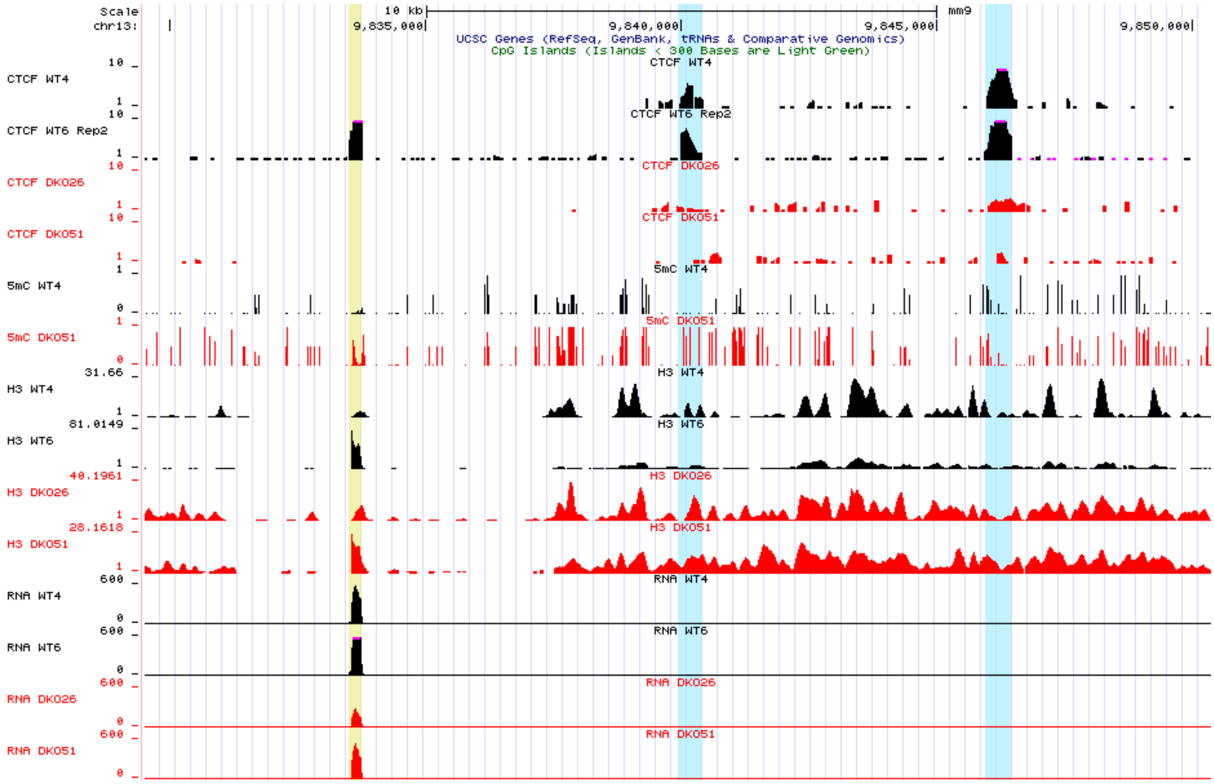

B

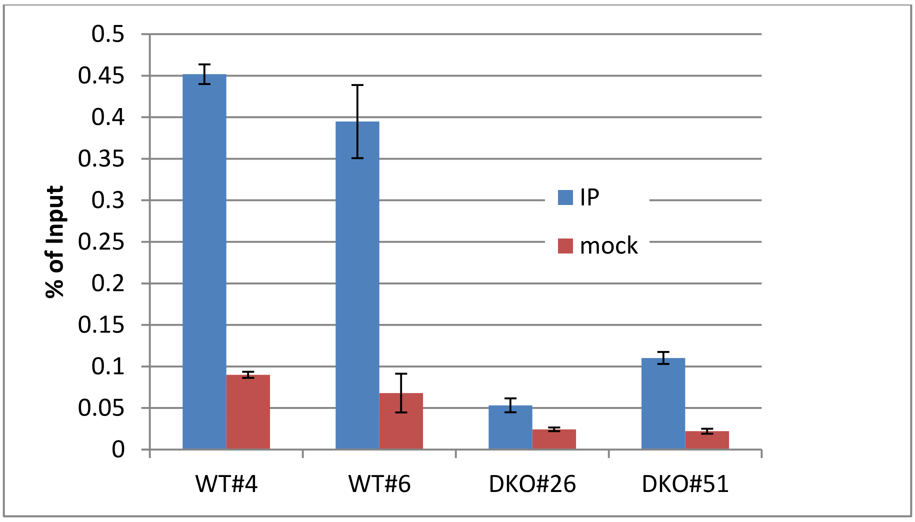

Figure S13. (A) Example region on chromosome 13 showing tracks of raw signals of CTCF and H3 ChIP-seq (in wild-type cells WT4 and WT6 and in TET knockout cells DKO26 and DKO51) and whole genome bisulfite sequencing and RNA-seq in WT4 and DKO51 cells. Highlighted rectangles show regions of pronounced CTCF binding changes. (B) qPCR validation of CTCF binding loss in DKO for the peak shown above at position chr13:9840011-9840567. Highlighted rectangles show regions of pronounced CTCF binding changes.

A

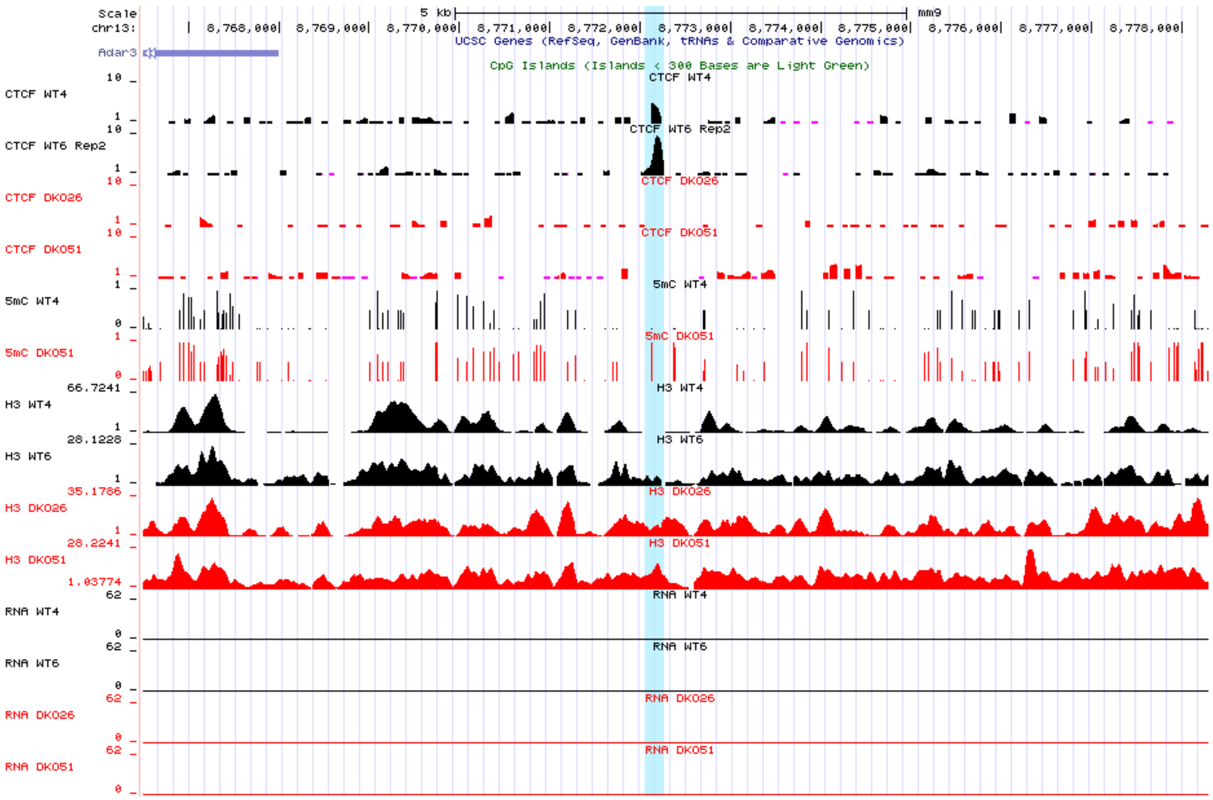

B

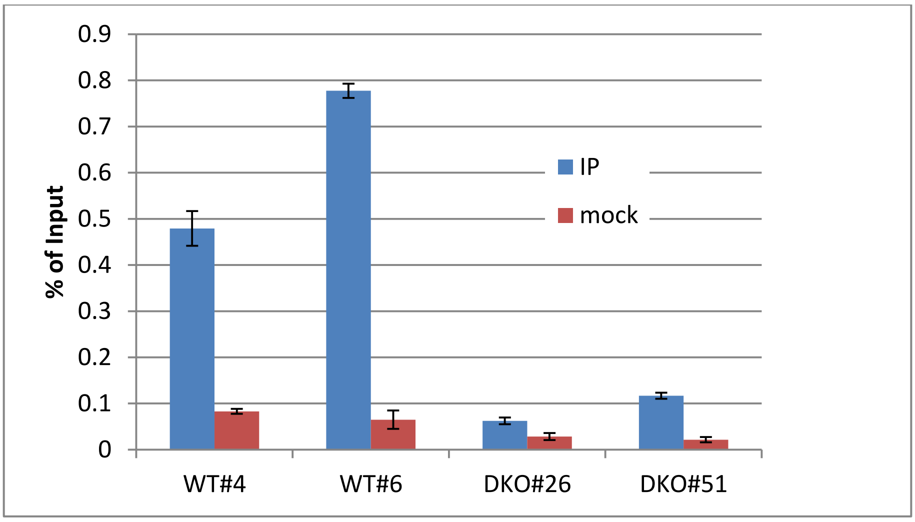

Figure S14. (A) Example region on chromosome 13 showing tracks of raw signals of CTCF and H3 ChIP-seq (in wild-type cells WT4 and WT6 and in TET knockout cells DKO26 and DKO51) and whole genome bisulfite sequencing and RNA-seq in WT4 and DKO51 cells. Highlighted rectangles show regions of pronounced CTCF binding changes. (B) qPCR validation of CTCF binding loss in DKO for the peak shown above at position chr13:8771848- 8772422.

A

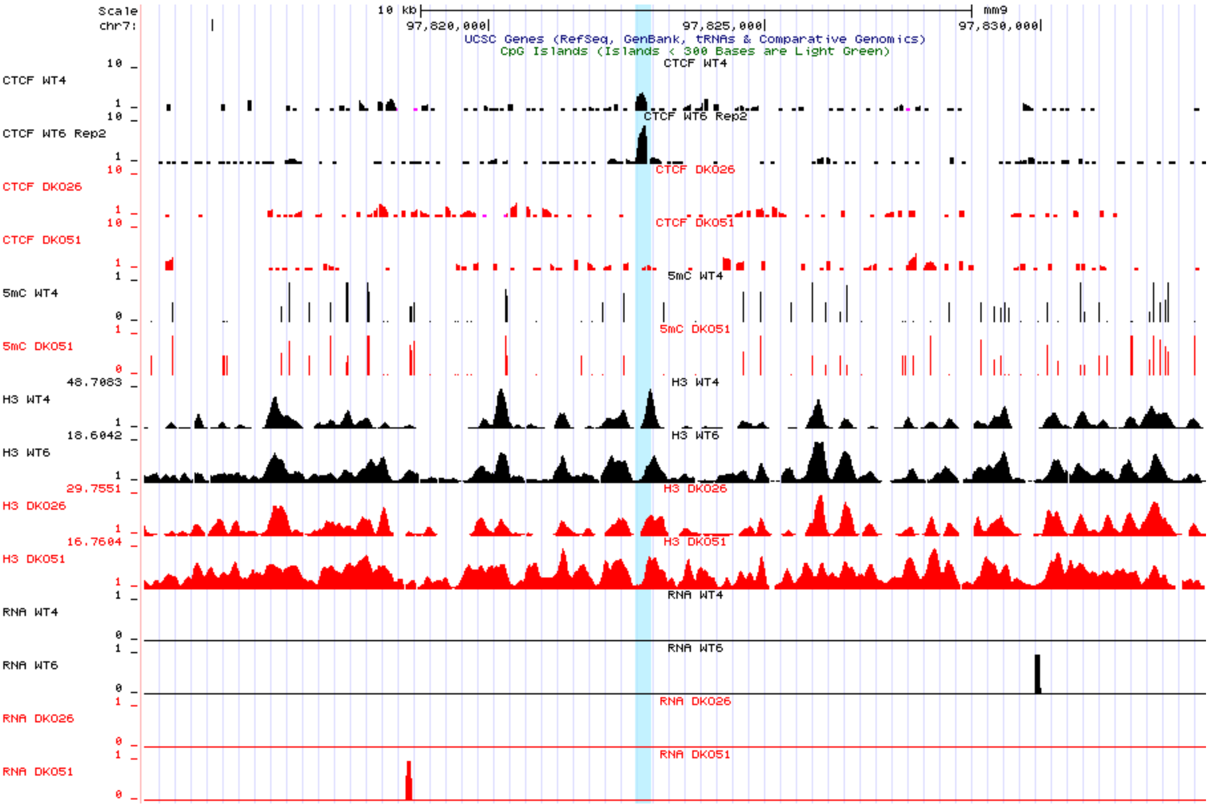

B

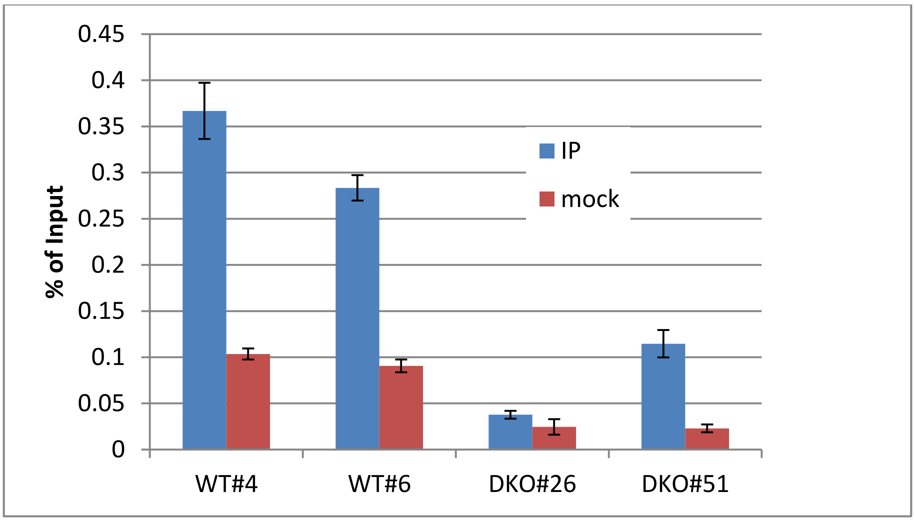

Figure S15. (A) Example region on chromosome 7 showing tracks of raw signals of CTCF ChIP-seq (in wild-type cells WT4 and WT6 and in TET knockout cells DKO26 and DKO51) and whole genome bisulfite sequencing in WT4 and DKO51 cells. Highlighted rectangles show regions of pronounced CTCF binding changes. (B) qPCR validation of CTCF binding loss in DKO for the peak shown above at position chr7:97822674-97823006.

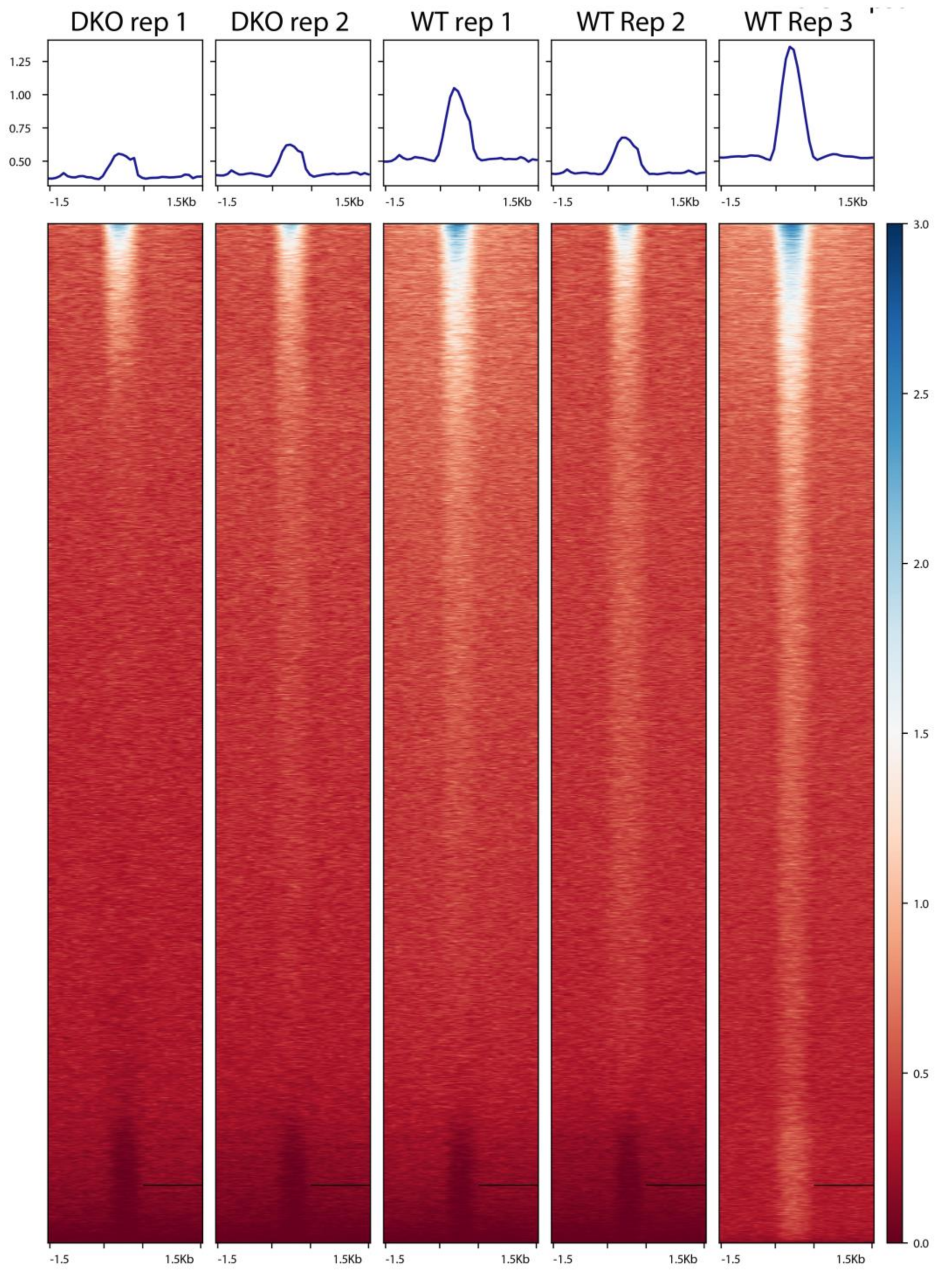

Figure S16. Average profiles and heatmaps of CTCF occupancy in DKO (two replicates, left) and WT (three replicates, right) around regions bound by TET1 in WT ESCs. The same genomic regions as in Figure S4 are used for this calculation.

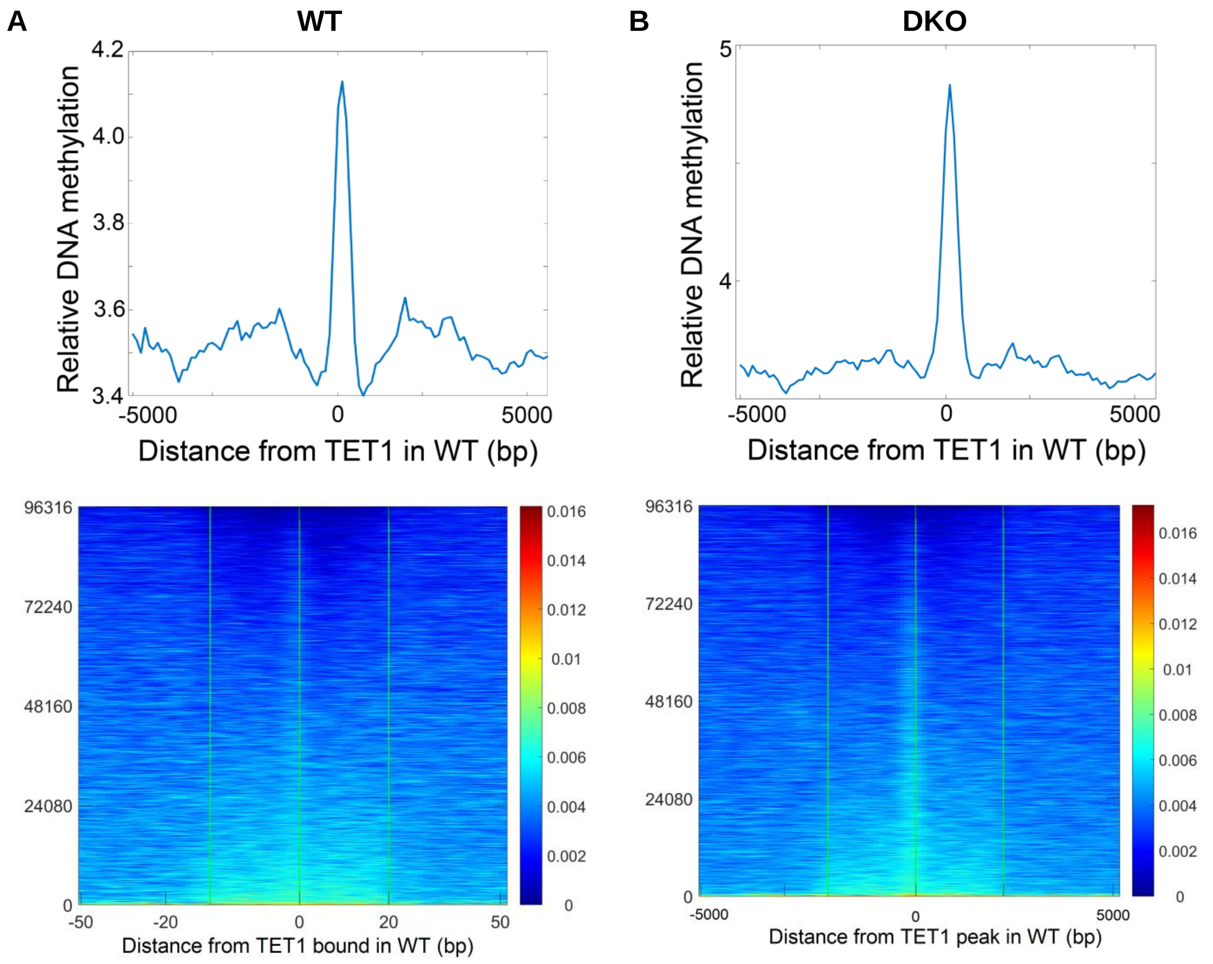

Figure S17. Average profiles and heatmaps of DNA methylation in WT (A) and DKO (B) around regions bound by TET1 in WT ESCs. The same TET1-bound genomic regions as in Figure S4 and S16 are used for this calculation.

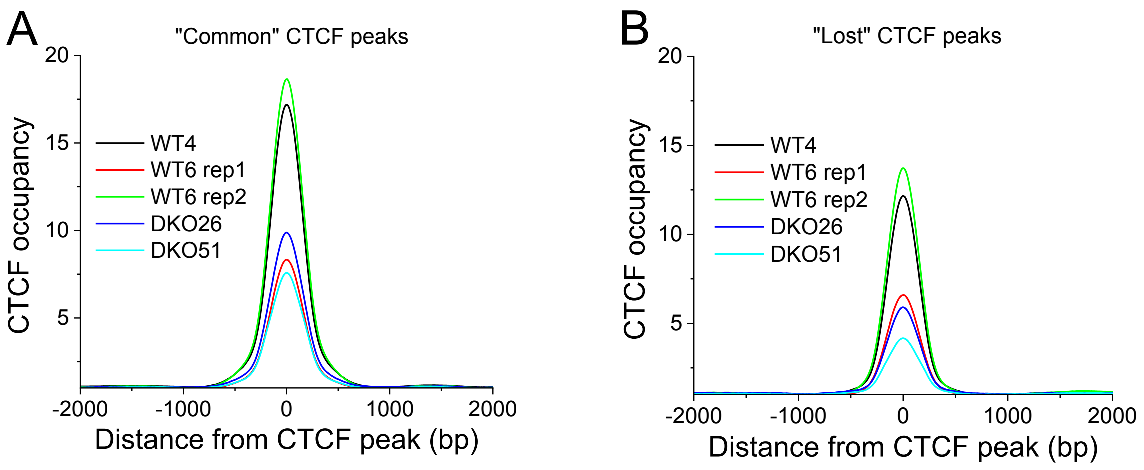

Figure S18. CTCF occupancy profiles around common (A) and lost (B) CTCF sites shown for each of the five CTCF ChIP-seq experiments prior to normalisation and averaging. Panels A and B of Figure 4 in the main manuscript have been calculated by averaging the corresponding replicates shown here and normalising by the averaged peak height of the common sites.

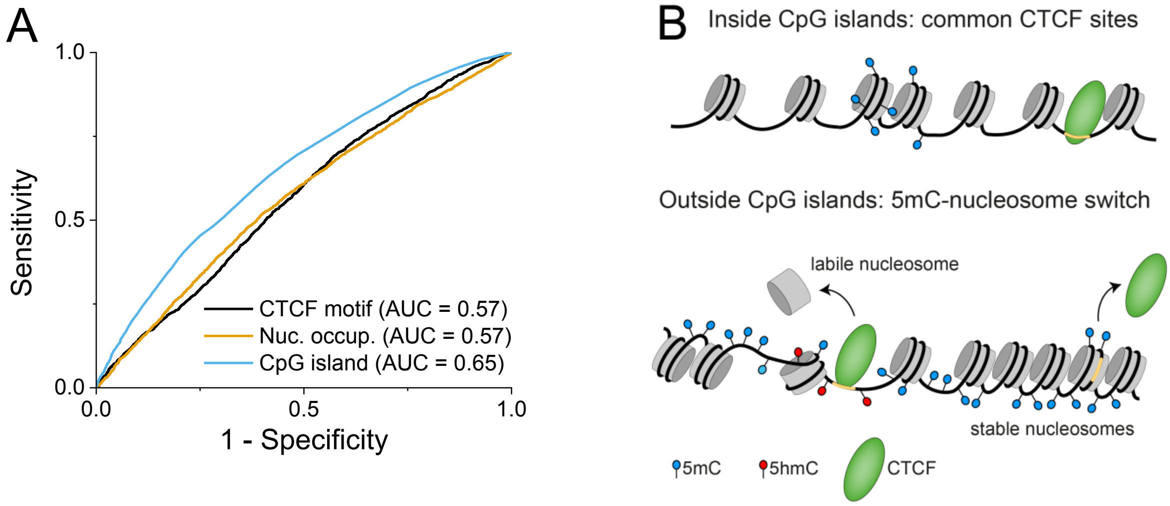

Figure S19. (A) Receiver-operator curves (ROC) for the prediction of CTCF loss in DKO cells taking into account only the sequence of the CTCF motif (black), nucleosome occupancy of 100-bp regions surrounding CTCF sites (brown) and CpG density in 1kb region surrounding CTCF sites (blue). (B) A schematic drawing outlining the possible mechanisms involved in CTCF binding to its motif. Inside CpG islands the nucleosome density is low and most CTCF sites are common. Outside CpG islands CTCF has to outcompete nucleosomes, which is regulated by DNA methylation. Most CTCF sites are not regulated directly by changes of DNA methylation inside the CTCF motif, but rather indirectly e.g. through changes in methylation-dependent nucleosomes.

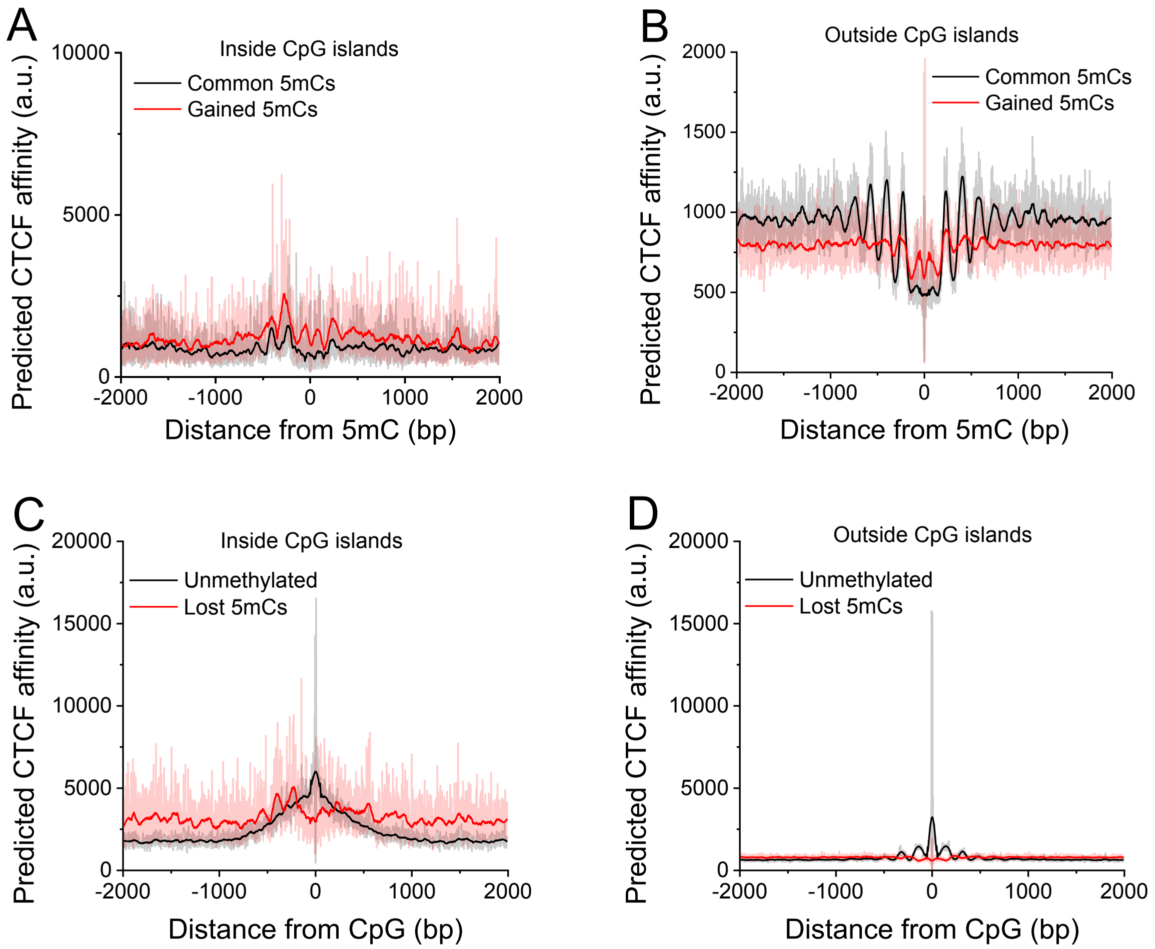

Figure S20. CTCF-DNA binding affinity predicted from the DNA sequence as a function of distance from CpGs. Calculations performed for four classes of CpGs, separately inside (A and C) and outside CpG islands (C and D). A and B) CpGs that are commonly methylated in both cell states (black) (methylation >0.8 both in WT and DKO) and those that gained methylation (<0.2 in WT and >0.5 in DKO) (red). C and D) Unmethylated CpGs (<0.2 in both WT and DKO) (black) and those that lost methylation (>0.5 in WT and <0.2 in DKO) (red).

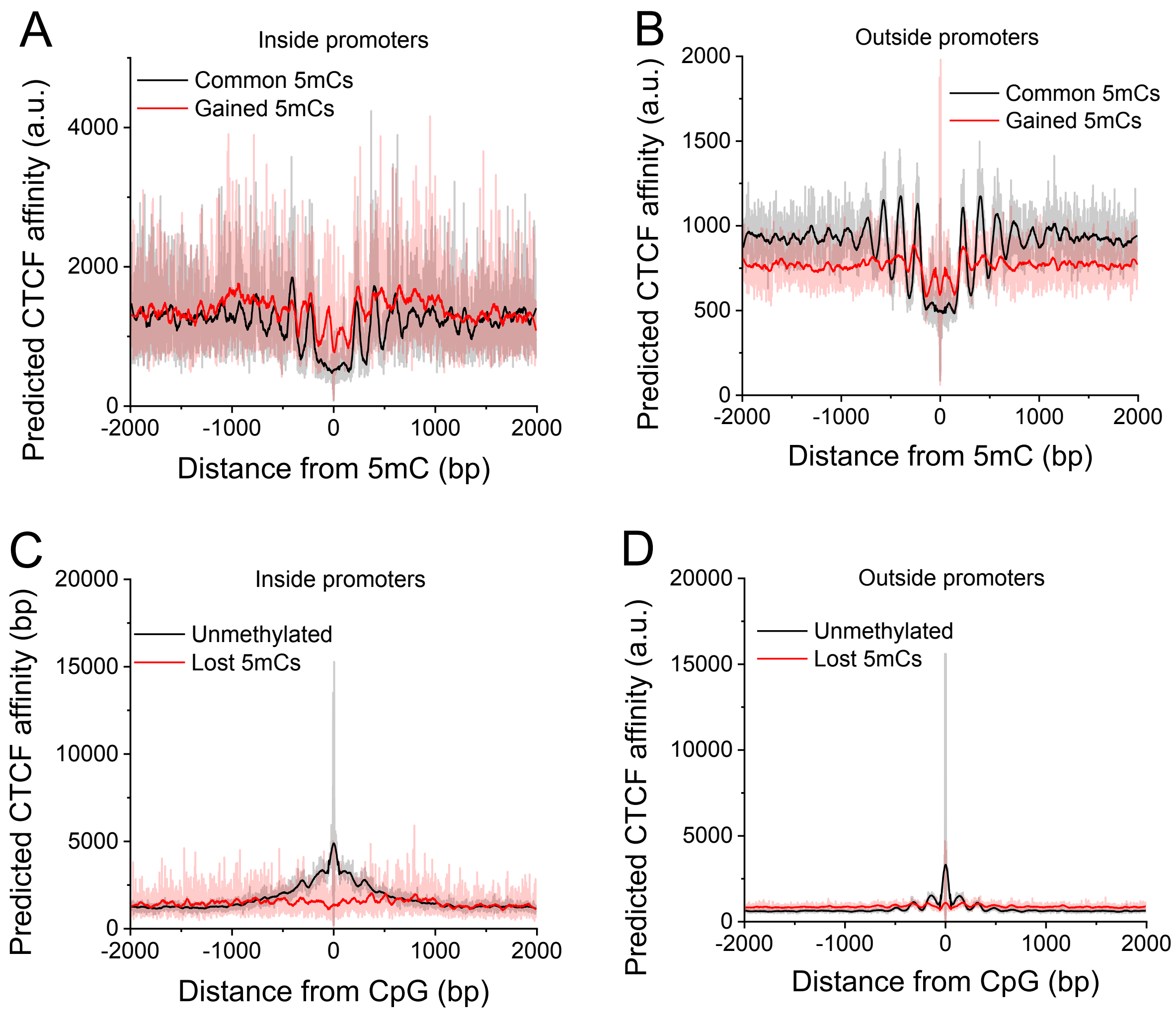

Figure S21. CTCF-DNA binding affinity predicted from the DNA sequence as a function of distance from CpGs. Calculations performed for four classes of CpGs, separately inside (A and C) and outside of promoters defined based on FANTOM5 CAGE data (C and D). A and B) CpGs that are commonly methylated in both cell states (black) (methylation >0.8 both in WT and DKO) and those that gained methylation (<0.2 in WT and >0.5 in DKO) (red). C and D) Unmethylated CpGs (<0.2 in both WT and DKO) (black) and those that lost methylation (>0.5 in WT and <0.2 in DKO) (red).

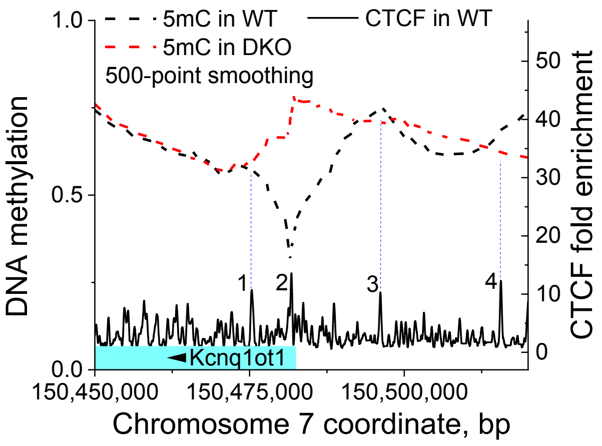

Figure S22. **Example genomic region on chromosome 7 showing the average DNA methylation patterns.** DNA methylation profiles have been smoothed with a 500-bp sliding window, separately for WT and DKO cells. Thick dashed lines show average 5mC level per CpG in WT (black) and DKO cells (red). Solid black line shows CTCF occupancy in WT cells. Blue dashed lines connect the peaks of CTCF occupancy with the corresponding extremes of the DNA methylation landscape. The light blue rectangle shows the gene body. The arrow indicates direction of transcription. Note that the heights of CTCF peaks on this figure are much smaller than those in the analogous Figure 6B in the main manuscript, yet the effect observed here is the same as in Figure 6B for 10-fold higher peaks.

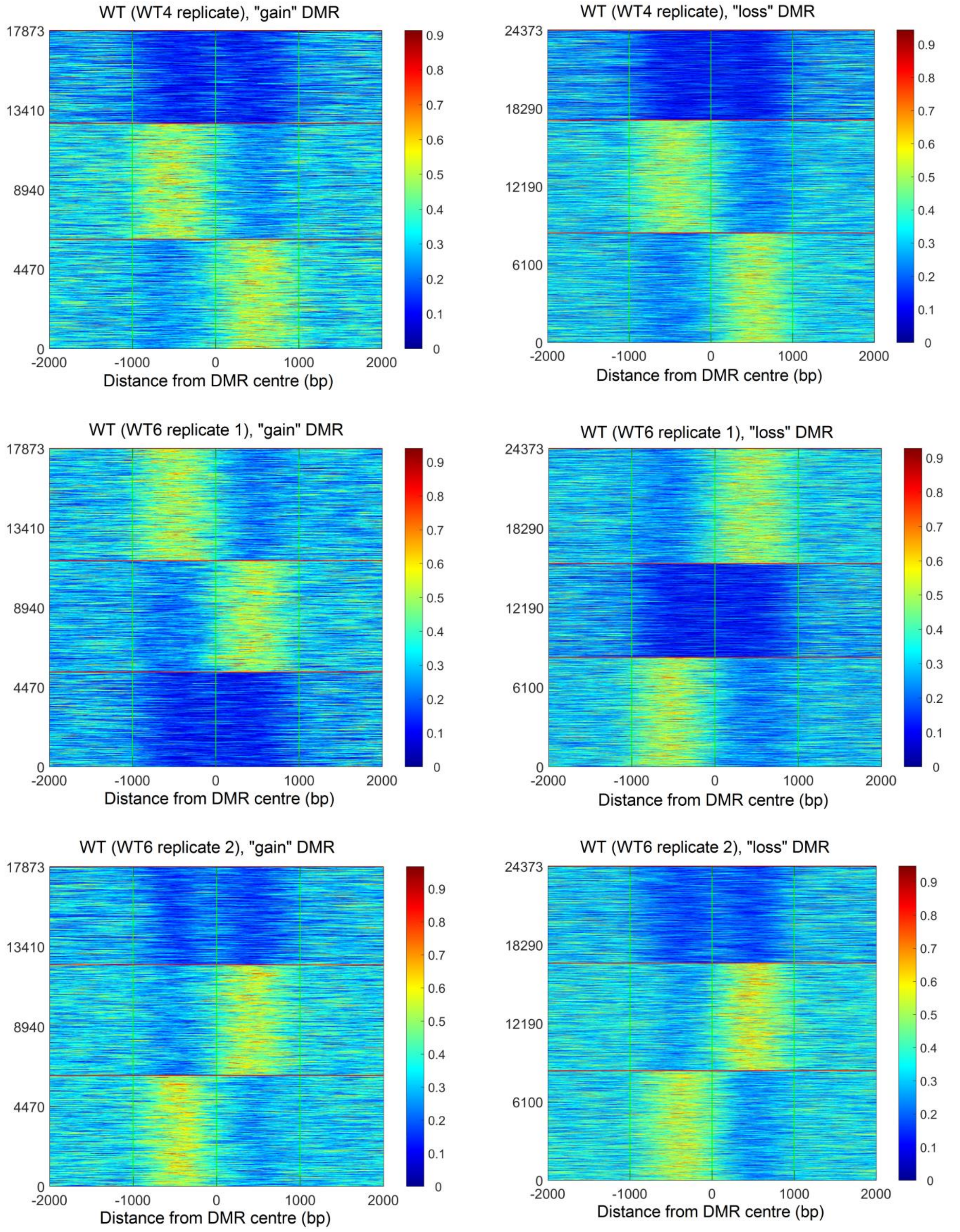

Figure S23. Heatmaps of experimental CTCF occupancy around centers of 5mC “gain” and “loss” DMRs in WT cells. *K*-means clustering with k=3 is performed using NucTools. Three replicate experiments have been conducted including two biological replicates (denoted WT4 and WT6 on the figure) and one additional technical replicate in WT6 cells. Heatmaps have been constructed separately for CTCF occupancy from each replicate experiment.

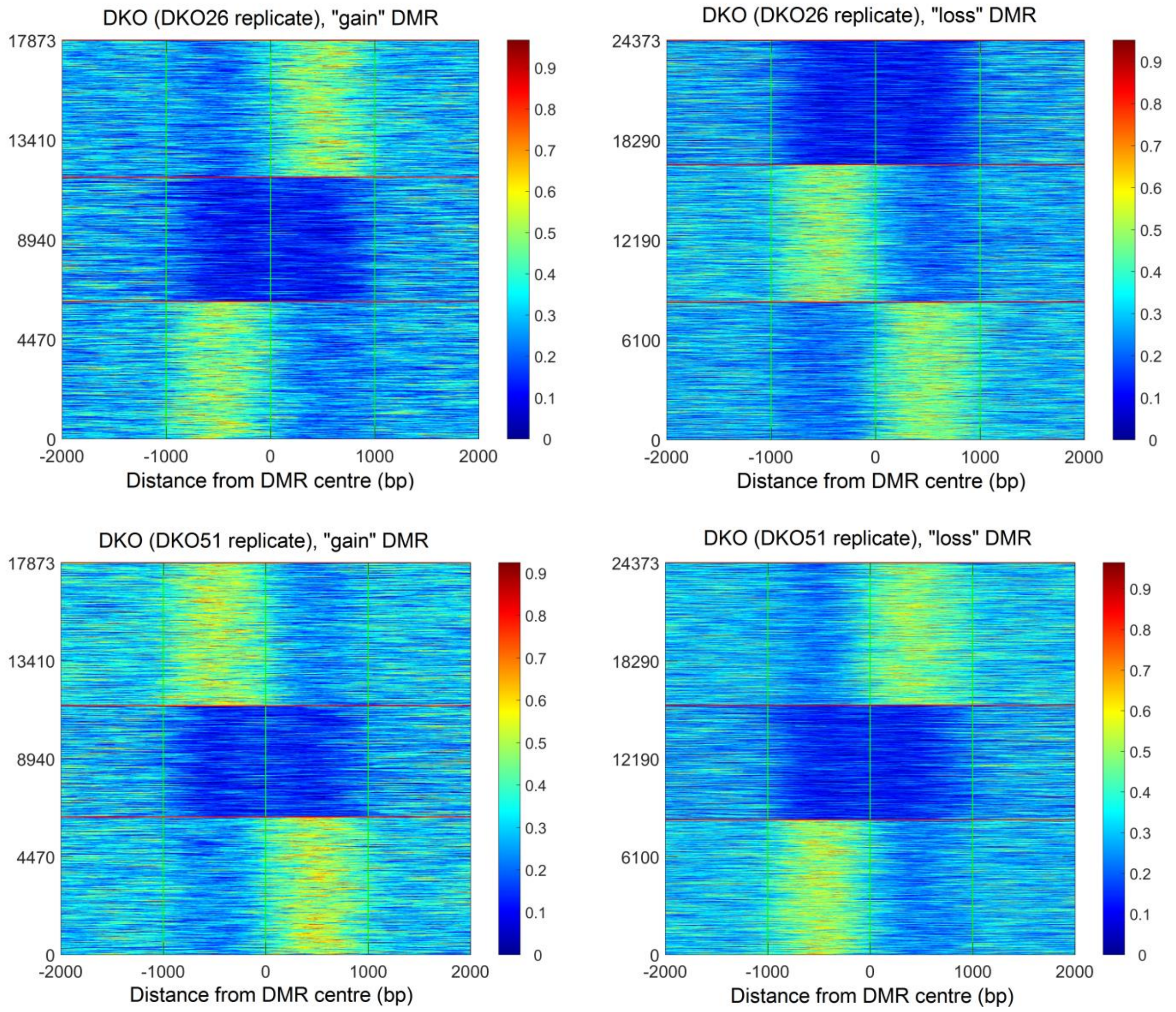

Figure S24. Heatmaps of experimental CTCF occupancy around centers of 5mC “gain” and “loss” DMRs in DKO cells. *K*-means clustering with k=3 is performed using NucTools. Two replicate experiments have been conducted including two biological replicates (denoted DKO26 and DKO51 on the figure). Heatmaps have been constructed separately for CTCF occupancy from each replicate experiment.

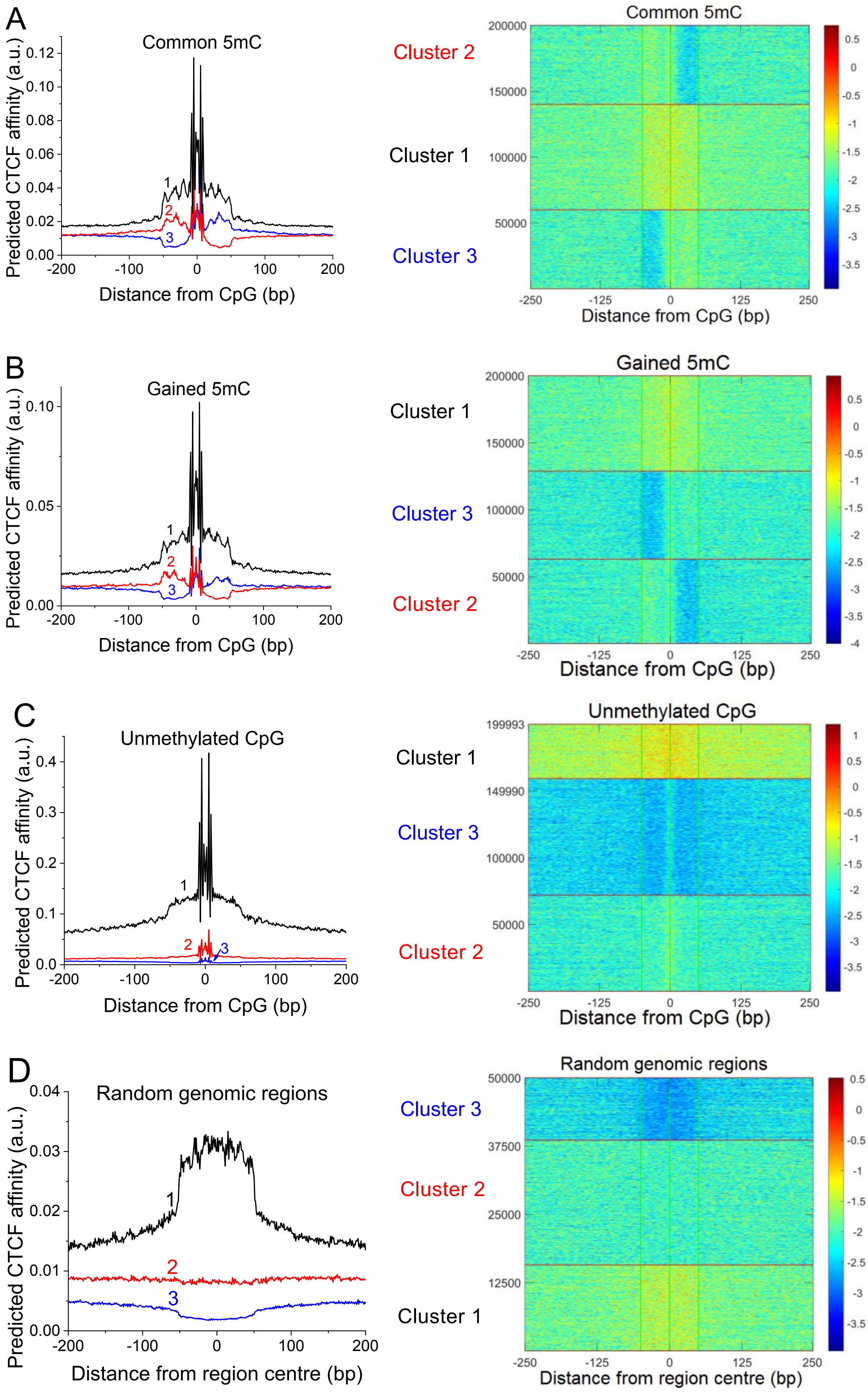
A
Common 5mC
Predicted CTCF affinity (a.u.)
Distance from CpG (bp)
Cluster 2
Cluster 1
Cluster 3
B
Gained 5mC
Cluster 1
Cluster 3
Cluster 2
C
Unmethylated CpG
Cluster 1
Cluster 3
Cluster 2
D
Random genomic regions
Distance from region centre (bp)
Cluster 3
Cluster 2
Cluster 1

Figure S25. *K*-means clustering of regions around four classes of CpGs based on the predicted CTCF affinity. In analogy to Figure 5, we included in the analysis commonly methylated CpGs (A), gained 5mCs (B), commonly unmethylated CpGs (C), as well as a similarly sized set of random genomic regions (D). In order to see the patterns instead of the raw CTCF affinity values, we plotted the logarithm of the affinity (which is linearly related to the intrinsic CTCF-DNA binding energy). *K*-means clustering was performed with k=3 based on CTCF occupancy within the interval [-50bp, +50bp] from the region center. The left panels show the average CTCF affinity profiles for each of the three identified clusters (k=3). The right panels show the heatmap of predicted CTCF affinity for all regions included in this calculation numbered along the Y axis (higher colour intensity corresponds to larger affinity). The red, black and light blue lines on the left panels show the three different clusters. This colour code is not related to the colour code in the heatmaps. For common (A) and gained 5mCs (B) three clusters can be seen: one symmetric cluster with higher affinities showing regular oscillations, and two asymmetric clusters with lower affinities on the left/right sides of the CpG. For commonly unmethylated CpGs (C) three symmetric clusters with different CTCF affinities can be seen; the oscillations of CTCF affinity are not observed in this case, unlike (A) and (B). Random genomic regions (D) have CTCF affinity of about one order of magnitude smaller that regions in (A-C), and have no oscillatory patterns.

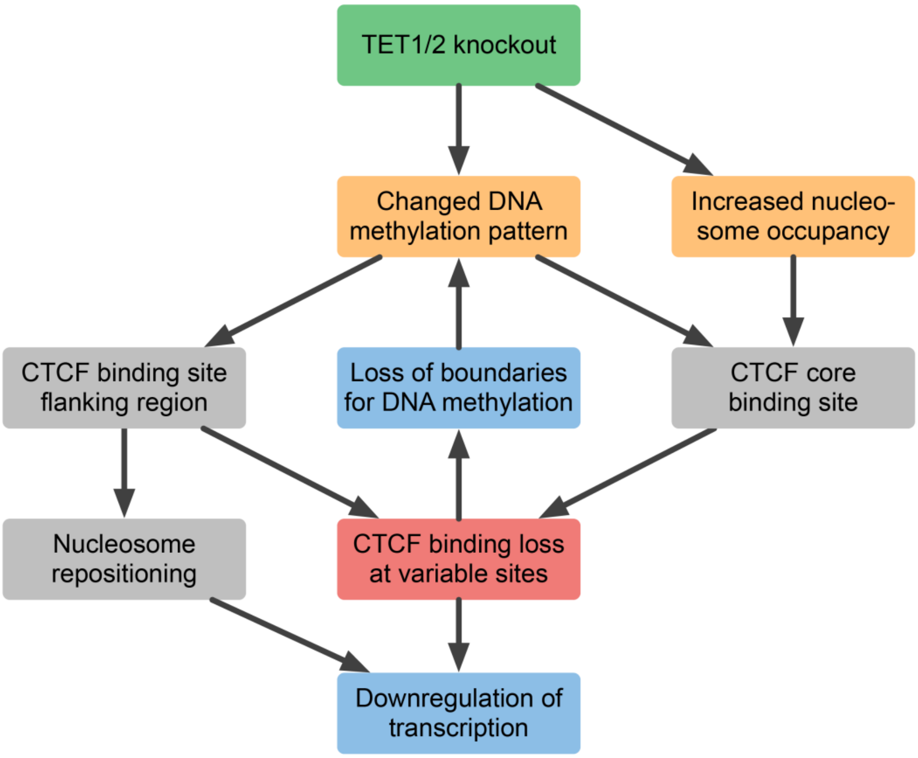

Figure S26. A scheme depicting potential molecular events connecting the methylation/nucleosome/CTCF switch. As a direct consequence of TET1/2 knockout there is major gain of DNA methylation and overall increase of nucleosome occupancy (10-fold more regions experienced increase of nucleosome occupancy than decrease of nucleosome occupancy). Increased nucleosome occupancy affects CTCF binding to its core site through histone/CTCF competition. DNA methylation changes affecting CTCF binding can be split into those happening inside the core CTCF motif (minority of cases) and those outside of CTCF binding sites (majority of cases). In the latter case DNA methylation affects CTCF binding through the methylation-dependent nucleosome rearrangement. CTCF loss determined by the changes of the methylation and chromatin landscape in turn can affect the spreading of DNA methylation. Furthermore, CTCF loss leads to the changes of gene expression which may in turn affect the upstream events.

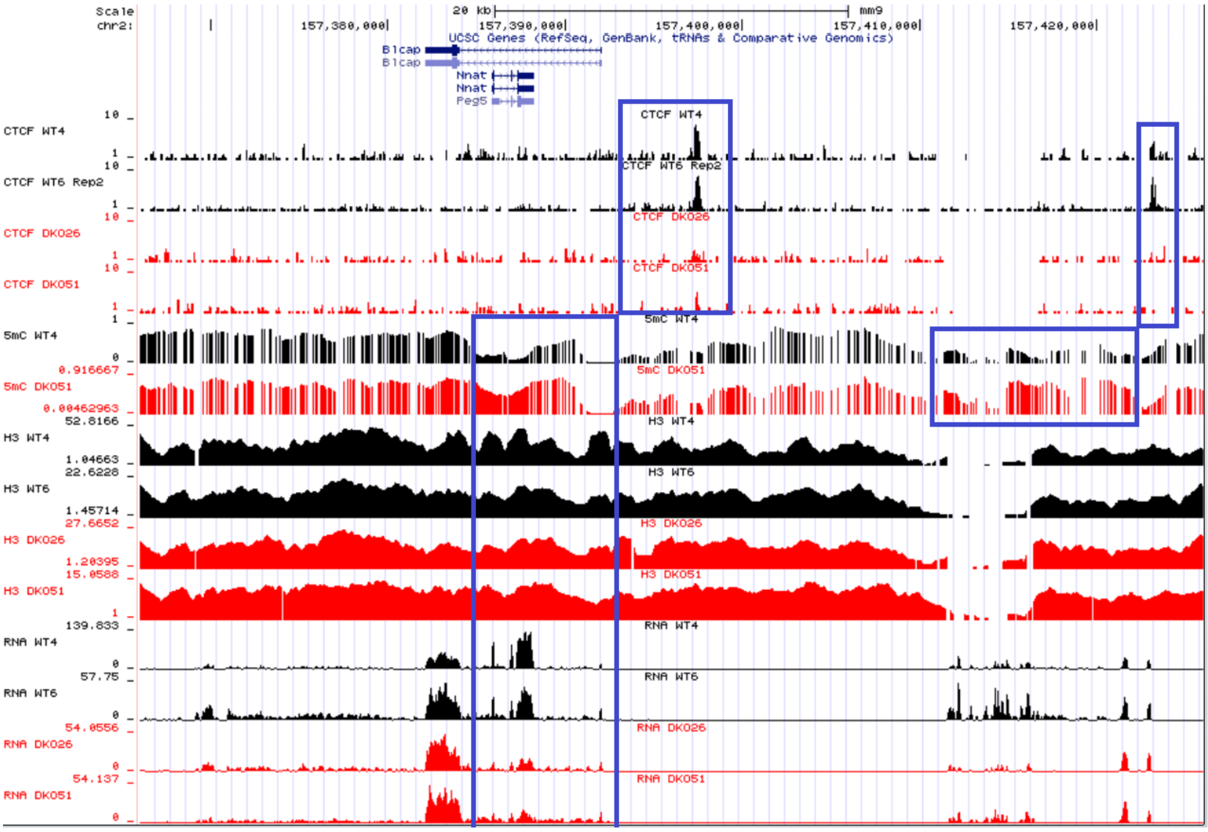

Figure S27. Genome browser tracks showing ChIP-seq for CTCF and histone H3, as well as WGBS and RNA-seq for an example region on Chromosome 2. The region includes a downregulated gene *Nnat* (log2 fold change -2.16; $p = 2.9 \times 10^{-7}$), which is located near lost CTCF sites at a loop boundary and is characterised by the change of DNA methylation in prolonged regions. Datasets corresponding to wildtype cells are in black and those corresponding to DKO cells are in red colour. Blue rectangles show regions of pronounced DNA methylation increase.

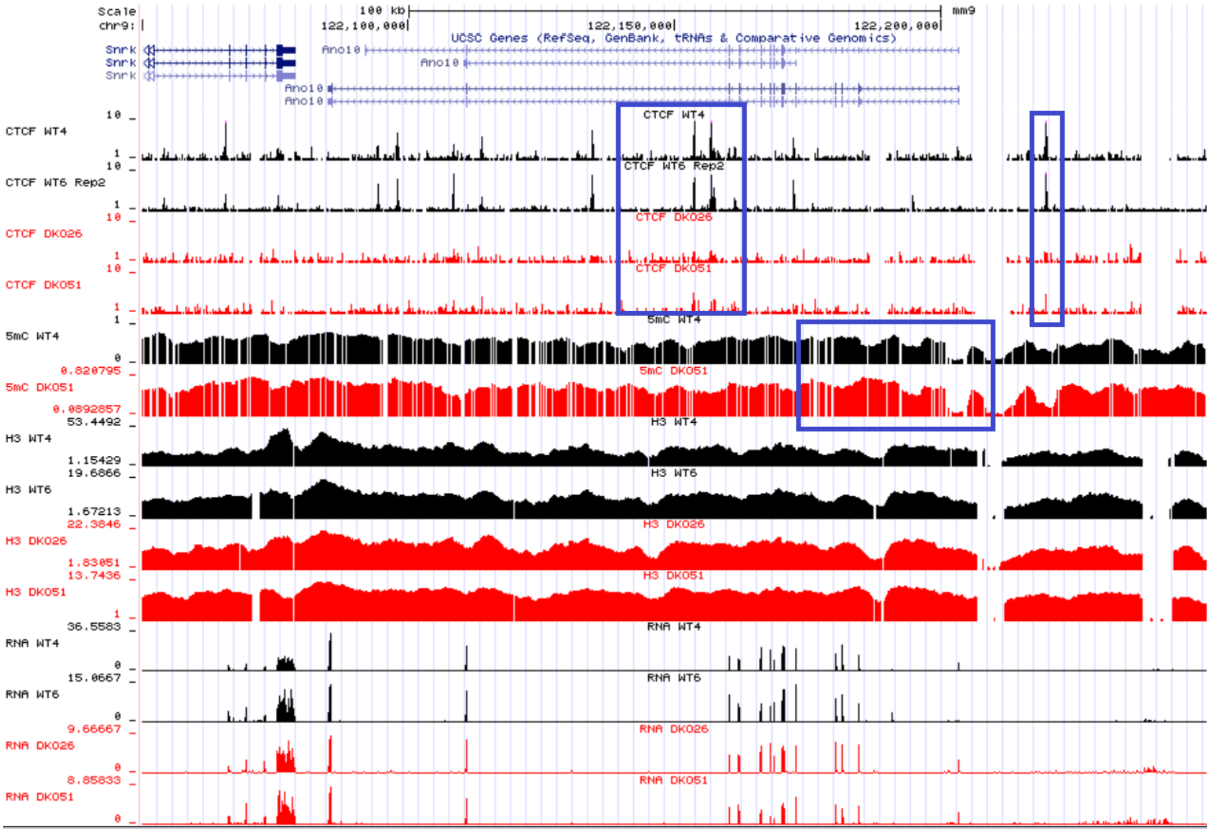

Figure S28. Genome browser tracks showing ChIP-seq for CTCF and histone H3, as well as WGBS and RNA-seq for an example region on Chromosome 9. The region includes a downregulated gene *Ano10* (log2 fold change -1.31; $p = 8 \times 10^{-4}$), which is located near several lost CTCF sites and is characterised by the change of DNA methylation in prolonged regions. Datasets corresponding to wildtype cells are in black and those corresponding to DKO cells are in red colour. Blue rectangles show regions of pronounced epigenome changes.

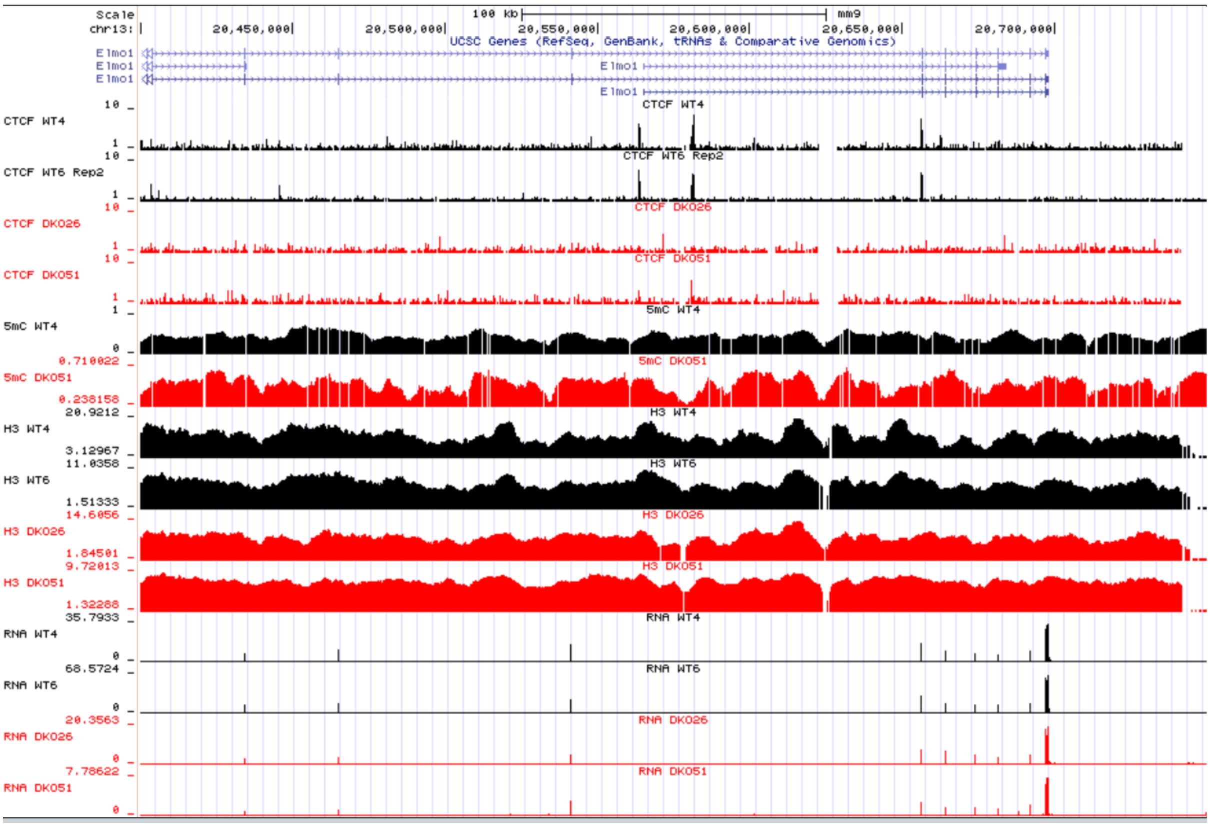

Figure S29. Genome browser tracks showing ChIP-seq for CTCF and histone H3, as well as WGBS and RNA-seq for an example region on Chromosome 13. Datasets corresponding to wildtype cells are in black and those corresponding to DKO cells are in red colour. This region shows large-scale changes of DNA methylation, loss of CTCF binding and downregulation of *Elmol* gene.

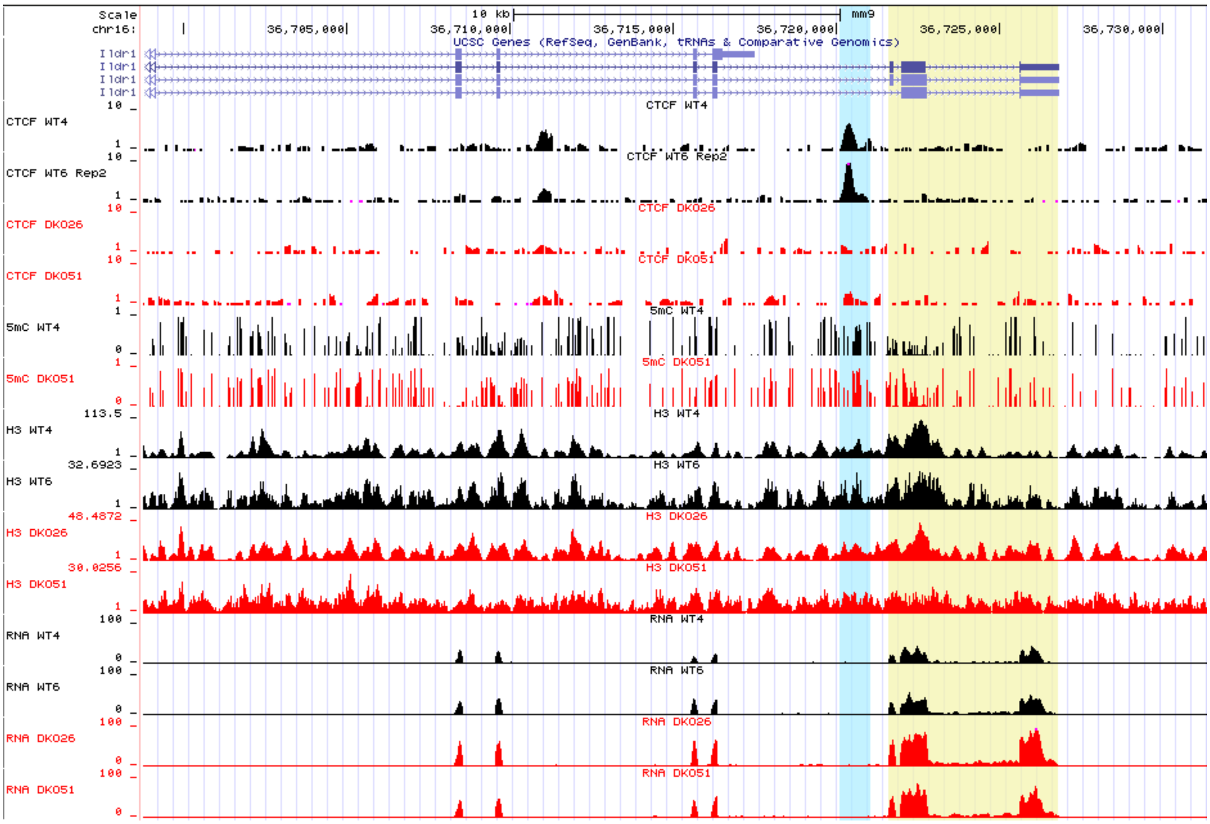

Figure S30. Genome browser tracks showing ChIP-seq for CTCF and histone H3, as well as WGBS and RNA-seq for an example region on Chromosome 16. Datasets corresponding to wildtype cells are in black and those corresponding to DKO cells are in red colour. The highlighted rectangles show the regions of pronounced epigenomic changes.

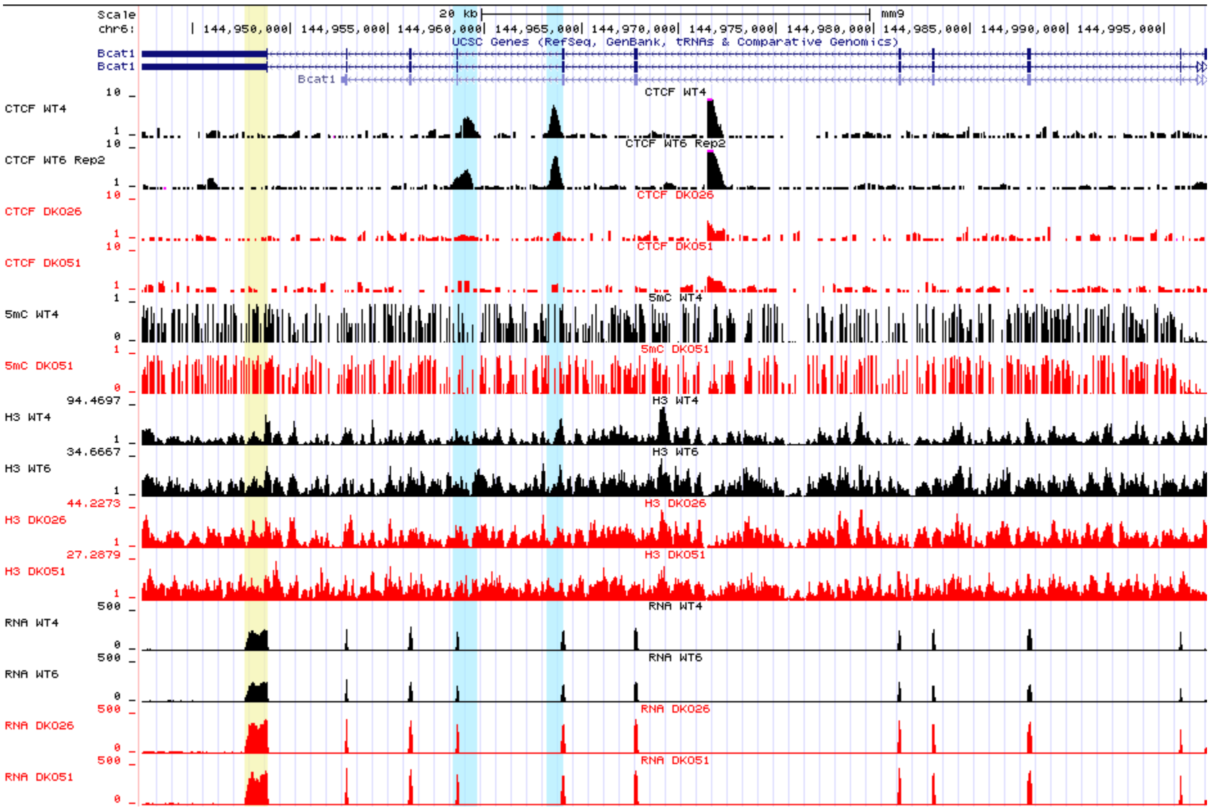

Figure S31. Genome browser tracks showing ChIP-seq for CTCF and histone H3, as well as WGBS and RNA-seq for an example region on Chromosome 6. Datasets corresponding to wildtype cells are in black and those corresponding to DKO cells are in red colour. The highlighted rectangles show the regions of pronounced epigenomic changes.

Table ST1. Pathways associated with regions that lost nucleosomes in DKO, calculated using Enrichr:

Index	Name	P-value	Adjusted p-value	Z-score	Combined score
1	PluriNetWork_Mus musculus_WP1763	0.008656	0.5657	-2.16	10.28
2	Interactome of polycomb repressive complex 2 (PRC2) _Homo sapiens_WP2916	0.006976	0.5657	-2.06	10.25
3	Amyotrophic lateral sclerosis (ALS)_Homo sapiens_WP2447	0.01078	0.5657	-2.00	9.05
4	TGF Beta Signaling Pathway_Mus musculus_WP113	0.01436	0.5657	-2.07	8.77
5	Wnt Signaling Pathway NetPath_Mus musculus_WP539	0.01693	0.5657	-1.82	7.44
6	Oxidative Stress_Homo sapiens_WP408	0.01780	0.5657	-1.84	7.43
7	Cardiac Progenitor Differentiation_Homo sapiens_WP2406	0.01585	0.5657	-1.79	7.42
8	TGF-beta Receptor Signaling_Homo sapiens_WP560	0.02098	0.5657	-1.92	7.40
9	BDNF signaling pathway_Homo sapiens_WP2380	0.02853	0.5657	-1.92	6.83
10	p38 MAPK Signaling Pathway_Homo sapiens_WP400	0.02580	0.5657	-1.71	6.25

Table ST2. TF binding associated with regions that lost nucleosomes in DKO, calculated using EnrichR:

Index	Name	P-value	Adjusted p-value	Z-score	Combined score
1	EGR1 (mouse)	0.001621	0.4832	-1.71	10.97
2	SP1 (mouse)	0.003503	0.5220	-1.63	9.20
3	TBX5 (human)	0.04086	1.000	-2.15	6.87
4	Sox17 (mouse)	0.04333	1.000	-1.89	5.94
5	E2F6 (human)	0.06980	1.000	-1.67	4.44
6	TEAD2 (mouse)	0.07805	1.000	-1.67	4.26
7	NFATC3 (human)	0.1267	1.000	-1.87	3.86
8	REPIN1 (human)	0.1037	1.000	-1.69	3.83
9	SMAD3 (human)	0.1664	1.000	-1.95	3.50
10	SMAD4 (mouse)	0.1274	1.000	-1.58	3.26

Table ST3. Differential gene expression.

Provided as a separate gzipped tab-separated text file.

Table ST4. Pathways associated with regions that gained nucleosomes in DKO, calculated using EnrichR:

Index	Name	P-value	Adjusted p-value	Z-score	Combined score
1	Nucleotide excision repair_Homo sapiens_hsa03420	0.02876	1.000	-1.86	6.60
2	Protein export_Homo sapiens_hsa03060	0.02574	1.000	-1.64	5.99
3	Cysteine and methionine metabolism_Homo sapiens_hsa00270	0.07271	1.000	-1.81	4.73
4	Glycosylphosphatidylinositol(GPI)-anchor biosynthesis_Homo sapiens_hsa00563	0.1270	1.000	-1.35	2.78
5	Complement and coagulation cascades_Homo sapiens_hsa04610	0.2025	1.000	-1.66	2.65
6	Protein processing in endoplasmic reticulum_Homo sapiens_hsa04141	0.2260	1.000	-1.62	2.41
7	Homologous recombination_Homo sapiens_hsa03440	0.1750	1.000	-1.35	2.35
8	Amoebiasis_Homo sapiens_hsa05146	0.2336	1.000	-1.59	2.32
9	Propanoate metabolism_Homo sapiens_hsa00640	0.2138	1.000	-1.37	2.12
10	Selenocompound metabolism_Homo sapiens_hsa00450	0.2077	1.000	-1.16	1.82

Table ST5. TF binding associated with regions that gained nucleosomes in DKO, calculated using EnrichR:

Index	Name	P-value	Adjusted p-value	Z-score	Combined score
1	TBP (mouse)	0.0001287	0.03731	-1.55	13.85
2	HNF1B (mouse)	0.005800	0.7157	-2.32	11.95
3	Sox2 (mouse)	0.008352	0.7157	-1.68	8.02
4	POU2F1 (mouse)	0.009871	0.7157	-1.66	7.65
5	SRF (mouse)	0.01538	0.8922	-1.60	6.69
6	STAT1 (mouse)	0.05279	1.000	-2.00	5.87
7	NR3C1 (mouse)	0.07490	1.000	-1.61	4.18
8	IRF2 (mouse)	0.08020	1.000	-1.58	3.97
9	Gata1 (mouse)	0.08575	1.000	-1.56	3.82
10	Tal1::Gata1 (mouse)	0.2265	1.000	-1.93	2.87

Table ST6. Gene Ontology at promoters that gained nucleosomes and were upregulated in DKO (log2 expression fold change > 1)

Annotation Cluster 1	Enrichment Score: 4.52	Count	P_Value	Benjamini
UP_KEYWORDS	Meiosis	13	1.4E-6	2.2E-4
GOTERM_BP_DIRECT	meiotic cell cycle	13	3.4E-6	6.4E-3
GOTERM_BP_DIRECT	spermatogenesis	20	5.6E-3	5.9E-1
Annotation Cluster 2	Enrichment Score: 3.63	Count	P_Value	Benjamini
UP_SEQ_FEATURE	disulfide bond	95	1.2E-6	1.9E-3
UP_KEYWORDS	Glycoprotein	134	1.5E-6	1.6E-4
UP_SEQ_FEATURE	glycosylation site:N-linked (GlcNAc...)	121	6.0E-6	4.8E-3
UP_KEYWORDS	Disulfide bond	111	1.1E-5	6.5E-4
UP_SEQ_FEATURE	signal peptide	101	3.4E-4	8.7E-2
GOTERM_CC_DIRECT	extracellular region	62	1.8E-3	2.6E-1
UP_KEYWORDS	Secreted	59	2.7E-3	5.1E-2
UP_KEYWORDS	Signal	125	4.5E-2	2.9E-1
GOTERM_CC_DIRECT	extracellular space	41	2.6E-1	9.3E-1
Annotation Cluster 3	Enrichment Score: 3.53	Count	P_Value	Benjamini
INTERPRO	Ferritin, conserved site	5	6.0E-5	2.5E-2
INTERPRO	Ferritin/DPS protein domain	5	1.9E-4	5.2E-2
INTERPRO	Ferritin	5	1.9E-4	5.2E-2
INTERPRO	Ferritin- like diiron domain	5	1.9E-4	5.2E-2
GOTERM_BP_DIRECT	iron ion transpo	6	2.5E-4	8.9E-2
UP_KEYWORDS	Iron storage	5	2.6E-4	7.2E-3
INTERPRO	Ferritin-related	5	2.6E-4	5.4E-2
INTERPRO	Ferritin-like superfamily	5	4.6E-4	7.4E-2
GOTERM_MF_DIRECT	ferric iron binding	5	7.7E-4	1.1E-1
GOTERM_BP_DIRECT	intracellular sequestering of iron ion	4	2.1E-3	4.3E-1
Annotation Cluster 4	Enrichment Score: 3.01	Count	P_Value	Benjamini
UP_KEYWORDS	Immunity	27	4.2E-6	3.2E-4
GOTERM_BP_DIRECT	immune system process	25	2.7E-5	2.5E-2
UP_KEYWORDS	Innate immunity	12	2.9E-2	2.3E-1
GOTERM_BP_DIRECT	innate immune response	13	2.8E-1	1.0E0
Annotation Cluster 5	Enrichment Score: 2.89	Count	P_Value	Benjamini
UP_KEYWORDS	Differentiation	32	1.8E-4	5.5E-3
GOTERM_BP_DIRECT	cell differentiation	37	2.1E-4	9.2E-2
UP_KEYWORDS	Developmental protein	37	6.1E-3	8.8E-2
GOTERM_BP_DIRECT	multicellular organism development	38	1.2E-2	7.9E-1
Annotation Cluster 6	Enrichment Score: 2.64	Count	P_Value	Benjamini
UP_KEYWORDS	Myosin	11	1.5E-7	4.6E-5
GOTERM_CC_DIRECT	myosin complex	11	3.5E-7	1.1E-4
INTERPRO	Myosin head, motor domain	9	3.2E-6	2.6E-3
SMA	MYSc	9	6.1E-6	1.1E-3
GOTERM_MF_DIRECT	motor activity	11	1.9E-5	1.2E-2
UP_KEYWORDS	Motor protein	13	5.5E-5	2.1E-3

Annotation Cluster 1		Enrichment Score: 4.52	Count	P_Value	Benjamini
	UP_SEQ_FEATURE	domain:Myosin head-like	6	4.9E-4	1.1E-1
	INTERPRO	IQ motif, EF-hand binding site	9	1.2E-3	1.2E-1
	UP_SEQ_FEATURE	region of interest:Actin-binding	5	2.1E-3	3.1E-1
	GOTERM_CC_DIRECT	myofibril	6	2.5E-3	2.4E-1
	INTERPRO	Myosin-like IQ motif-containing domain	4	8.4E-3	2.8E-1
	INTERPRO	Myosin tail	4	9.8E-3	2.9E-1
	SMA	IQ	6	1.0E-2	2.4E-1
	KEGG_PATHWAY	Tight junction	9	1.6E-2	5.1E-1
	UP_KEYWORDS	Calmodulin-binding	9	1.8E-2	1.7E-1
	UP_SEQ_FEATURE	domain:IQ 2	4	2.2E-2	7.4E-1
	UP_SEQ_FEATURE	domain:IQ 1	4	2.4E-2	7.6E-1
	GOTERM_CC_DIRECT	brush border	6	3.7E-2	6.1E-1
	UP_SEQ_FEATURE	domain:IQ 3	3	4.1E-2	8.6E-1
	INTERPRO	Myosin, N-terminal, SH3-like	3	4.9E-2	6.9E-1
	GOTERM_MF_DIRECT	microfilament motor activity	3	5.5E-2	7.6E-1
	GOTERM_MF_DIRECT	calmodulin binding	9	7.3E-2	8.2E-1
	UP_KEYWORDS	Actin-binding	11	7.9E-2	4.2E-1
	GOTERM_MF_DIRECT	actin binding	11	3.0E-1	9.8E-1
	UP_SEQ_FEATURE	region of interest:Calmodulin-binding	3	3.2E-1	1.0E0
Annotation Cluster 7		Enrichment Score: 2.42	Count	P_Value	Benjamini
	UP_KEYWORDS	Eye lens protein	6	8.5E-5	2.9E-3
	GOTERM_MF_DIRECT	structural constituent of eye lens	6	2.3E-4	6.9E-2
	GOTERM_BP_DIRECT	eye development	7	3.9E-4	1.2E-1
	UP_SEQ_FEATURE	domain:Beta/gamma crystallin 'Greek key' 1	4	4.6E-3	4.6E-1
	UP_SEQ_FEATURE	domain:Beta/gamma crystallin 'Greek key' 2	4	4.6E-3	4.6E-1
	UP_SEQ_FEATURE	domain:Beta/gamma crystallin 'Greek key' 3	4	4.6E-3	4.6E-1
	UP_SEQ_FEATURE	domain:Beta/gamma crystallin 'Greek key' 4	4	4.6E-3	4.6E-1
	INTERPRO	Gamma-crystallin-related	4	7.1E-3	2.6E-1
	INTERPRO	Beta/gamma crystallin	4	7.1E-3	2.6E-1
	SMA	XTALbg	4	9.2E-3	2.4E-1
	UP_SEQ_FEATURE	region of interest:Connecting peptide	5	1.1E-2	6.4E-1
	UP_SEQ_FEATURE	site:Susceptible to oxidation	3	3.0E-2	8.0E-1
	GOTERM_BP_DIRECT	lens development in camera-type eye	4	6.9E-2	9.7E-1
Annotation Cluster 8		Enrichment Score: 2.36	Count	P_Value	Benjamini
	UP_KEYWORDS	Calcium	37	3.7E-4	9.5E-3
	INTERPRO	EF-hand domain	15	9.6E-4	1.1E-1

Table ST7. TF motifs enriched within 1,000 bp from DMR boundaries

Motifs found	TF matches	E-value
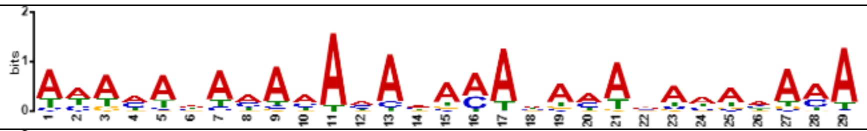	ZNF384 (MA1125.1) Mtf1 (UP00097_2)	9.9e-139
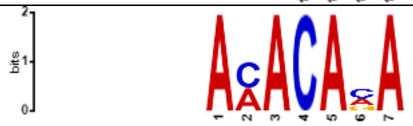	FOXP2 (MA0593.1)	3.4e-024
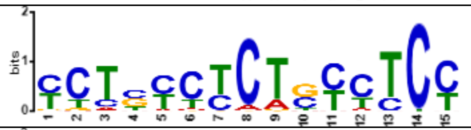	ZNF263 (MA0528.1)	1.0e-111
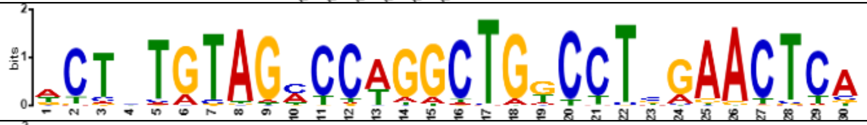	Nr5a2 (MA0505.1)	8.6e-106
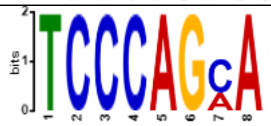	STAT3 (MA0144.2) Stat5a::Stat5b (MA0519.1)	1.7e-024
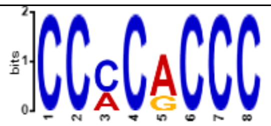	KLF5 (MA0599.1) Klf1 (MA0493.1) SP1_DBD	1.1e-014
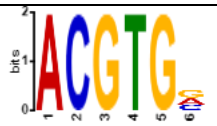	Mlxip (MA0622.1) USF1 (MA0093.2) TFE3_DBD	1.5e-004
